# Adversarial path planning for optimal CCTV surveillance: a case study on nuclear facility security optimization

**DOI:** 10.1038/s41598-026-47647-8

**Published:** 2026-04-17

**Authors:** Ahmed E. Salman, Noha Shaaban, W. I. Zidan, Mohamed H. Saad

**Affiliations:** 1 Operational Safety and Human Factors Department, Nuclear and Radiological Safety Research Center (NRSRC), Egyptian Atomic Energy Authority, Cairo, Egypt; 2 Nuclear Safeguards and Physical Protection Department, Nuclear and Radiological Safety Research Center (NRSRC), Egyptian Atomic Energy Authority, Cairo, Egypt; 3 Radiation Engineering Department, National Center for Radiation Research and Technology, Egyptian Atomic Energy Authority, Cairo, Egypt

**Keywords:** Adversarial path planning, CCTV optimization, Nuclear security, Surveillance coverage, Resource allocation, Intrusion detection, Engineering, Mathematics and computing

## Abstract

The security of critical infrastructure, particularly nuclear facilities, is paramount for public safety. Conventional Closed-Circuit Television (CCTV) surveillance relies on static camera placement, which fails to adapt to dynamic adversarial behavior, resulting in coverage gaps and inefficiencies. This study proposes a novel Adversarial Path Planning (APP) framework that integrates game theory, probabilistic risk assessment, and bilevel optimization to enhance surveillance coverage, intrusion detection, and resource allocation. APP simulates adversarial movement and iteratively refines camera placement to dynamically counter evolving threats, minimizing blind spots and optimizing detection probability. The framework models the facility as a weighted surveillance graph, identifying high-risk intrusion paths to optimize camera positioning for maximum coverage with minimal redundancy. A case study on a hypothetical nuclear power plant demonstrates APP’s effectiveness: it achieves 95% surveillance coverage, improves detection accuracy to 98%, and reduces dead zones by 85%, significantly outperforming conventional methods like the Genetic Algorithm (GA), Particle Swarm Optimization (PSO), and Ant Colony Optimization (ACO). Furthermore, APP reduces the required number of cameras by 40% while improving cost efficiency by 27%, underscoring its potential for resource-conscious security optimization. The findings establish APP as a scalable and computationally efficient solution adaptable to nuclear facilities, border surveillance, and other high-risk critical infrastructure. Future research should explore AI-driven real-time threat detection and autonomous surveillance systems to further enhance responsiveness in dynamic security environments.

**Supplementary Information:**

The online version contains supplementary material available at 10.1038/s41598-026-47647-8.

## Introduction

The security of critical infrastructure, particularly nuclear facilities, is of paramount importance to ensuring public safety, operational resilience, and protection against evolving adversarial threats. While nuclear safety culture has long been established to prevent accidental radioactive material leaks, nuclear security culture emerged later in response to intentional security breaches, sabotage risks, and adversarial attacks. Unlike safety systems, which aim to correct equipment failures and human errors, security systems must be proactively designed to anticipate, detect, and neutralize deliberate threats. Given the increasing complexity of modern security risks, surveillance strategies must integrate adaptive intelligence, real-time threat assessment, and dynamic response mechanisms to enhance intrusion prevention and facility protection^1^.

Closed-Circuit Television (CCTV) surveillance plays a fundamental role in physical security, providing continuous monitoring, intrusion deterrence, and real-time threat detection. However, traditional CCTV deployment strategies rely on static placement models that fail to account for adversarial behavior, leading to coverage gaps, inefficient resource allocation, and blind spots. Moreover, conventional surveillance planning often depends on manual camera placement, which is subjective, time-consuming, and suboptimal for high-security environments. These limitations necessitate a computationally optimized and dynamically adaptive approach to CCTV surveillance system design^2,3^.

This study aims to develop and validate the Adversarial Path Planning (APP) framework with the following specific objectives: (1) Algorithmic Formulation: To establish a novel bilevel optimization model that strategically integrates probabilistic adversarial path prediction with optimal static camera placement, addressing a gap between spatial coverage and dynamic threat response. (2) Empirical Validation: To demonstrate through comprehensive simulation that APP significantly outperforms established coverage-based and heuristic optimization methods—including Genetic Algorithm (GA), Particle Swarm Optimization (PSO), and Ant Colony Optimization (ACO)—in critical security metrics: surveillance coverage, detection probability, dead-zone reduction, and cost efficiency. (3) Practical Implementation: To provide a scalable, computationally efficient, and reproducible decision-support tool for the design-phase planning of robust CCTV surveillance networks in high-security facilities, using a standardized nuclear power plant layout as a case study.

The development and evaluation of the APP framework are based on the following foundational assumptions: (1) Adversary Model: Potential intruders are rational, risk-averse actors who select paths that minimize their exposure to surveillance. They are assumed to have perfect knowledge of the camera network’s coverage when planning their intrusion route. (2) Sensor Model: CCTV cameras are modeled with deterministic geometric and performance characteristics (field of view, resolution, range) based on standard specifications. Initial evaluations assume ideal operational conditions (unobstructed line-of-sight, optimal lighting). (3) Facility Model: The facility’s physical layout, including the location of critical assets, perimeter boundaries, and permanent structures, is known, accurate, and static during the security system design optimization phase. (4) Optimization Scope: The framework is designed for the strategic, *design-phase* placement of fixed surveillance assets. Real-time, post-deployment dynamic reconfiguration of hardware or active countermeasures is not within the current model’s scope.

Guided by these assumptions, the APP framework implements a systematic methodology. Effective CCTV surveillance requires strategic camera placement, adaptive response mechanisms, and AI integration to ensure comprehensive coverage and real-time threat detection^4–9^. The APP integrates game theory, risk assessment, and optimization to proactively anticipate threats and dynamically adjust camera placement. APP follows a multi-phase approach, beginning with comprehensive risk assessment to identify vulnerabilities and critical zones, forming the basis for strategic deployment^10–12^. The APP algorithm employs computational techniques for optimal placement, incorporating FOV/AOV analysis and dead-zone reduction for efficient coverage^12–14^. APP’s performance is validated using quantitative metrics: coverage percentage, detection accuracy, dead-zone reduction, and cost efficiency.

The experimental evaluation of the APP framework at the Lone Pine Nuclear Power Plant (LPNPP) demonstrates significant improvements over traditional static surveillance placement methods. The results confirm that APP achieves 95% coverage, 98% detection accuracy, and reduces dead zones by 85%, substantially outperforming optimization techniques such as Genetic Algorithm (GA), Particle Swarm Optimization (PSO), and Ant Colony Optimization (ACO). Additionally, the APP framework reduces the number of required cameras by 40%, leading to a 27% increase in cost efficiency, reinforcing its resource-conscious security optimization capabilities. These findings underscore the superiority of APP in high-security environments, demonstrating its effectiveness in adaptive, intelligent, and cost-efficient surveillance system deployment^15–20^.

Unlike traditional coverage optimization methods that treat camera placement statically, or game-theoretic approaches that focus on mobile assets, APP uniquely integrates adversarial path prediction with fixed camera optimization in a bilevel framework. This addresses the gap between spatial coverage maximization and dynamic threat response in high-security environments.

The main methodological contribution of this study lies in integrating adversarial path simulation with bilevel optimization for CCTV placement. Unlike traditional optimization approaches such as GA, PSO, or ACO that focus solely on coverage, the proposed APP framework explicitly models rational adversarial behavior and iteratively adapts surveillance deployment based on evolving intrusion paths, thereby enhancing detection performance and efficiency.

In summary, by systematically analyzing the interdependencies between security effectiveness factors, this study provides valuable insights for security professionals, policymakers, and facility managers, facilitating data-driven decision-making in high-risk surveillance planning. The proposed APP framework functions as a decision-support tool for enhancing security operations, mitigating intrusion risks, and optimizing surveillance resource allocation, ensuring a resilient and cost-effective security infrastructure in high-threat environments.

## Related work

### Traditional surveillance optimization

Early approaches to camera placement focused on static coverage maximization using geometric models^12,21^ and integer programming^22,23^. Heuristic methods such as Genetic Algorithms (GA)^19^, Particle Swarm Optimization (PSO)^10^, and Ant Colony Optimization (ACO)^24,25^ were later applied to large-scale facility layouts. These methods optimize coverage metrics but typically lack dynamic threat modeling components.

### AI-enhanced surveillance systems

Recent advances integrate artificial intelligence with surveillance optimization. Deep learning enables automated anomaly detection^26^, while computer vision improves threat recognition^27^. Machine learning approaches adapt camera parameters based on environmental conditions^28^, but often treat camera placement as a separate, static problem.

### Game-theoretic security frameworks

Adversarial security games, pioneered by Tambe et al.^29^, model intruder-defender interactions using Stackelberg game theory. These frameworks have been applied to randomized patrol scheduling^30^ and resource allocation^31^ but typically focus on mobile security assets rather than fixed camera optimization.

### Dynamic camera placement

Deep reinforcement learning (DRL) enables adaptive camera reconfiguration. Fu et al.^32^ used multi-agent DRL for dynamic camera scheduling in smart cities, while Sharma and Roy^33^ applied Q-learning for real-time camera adjustment. These approaches excel at temporal adaptation but often lack spatial optimization of initial placement.

### Contributions of this work

This paper makes the following key contributions, which advance the state of the art in surveillance system optimization for fixed critical infrastructure:

#### Novel bilevel optimization framework for static-dynamic coupling

Prior work typically separates spatial camera placement optimization (static) from adversarial behavior modeling (dynamic), often treating the latter as an external evaluation step. APP formulates these as a *single*,* unified bilevel optimization problem* (Eqs. 9–11), where the upper level (camera placement) explicitly reacts to the lower level (adversarial path planning) in an iterative feedback loop.

Distinction from prior work: Unlike game-theoretic security games^29–31^ that focus on scheduling mobile patrols, or DRL methods^32,33^ that adapt existing cameras in real-time, APP is designed for the *optimal initial placement of fixed assets* against an adaptive adversary, filling a gap in the security design phase.

#### Integrated probabilistic adversary model within the optimization loop

APP incorporates a *risk-sensitive*,* probabilistic movement model* (Eq. 13) directly into the optimization process. The adversary’s path choices are continuously updated based on the evolving surveillance layout via a dynamic risk update rule (Eq. 19), creating a co-adaptive simulation.

Distinction from prior work: This moves beyond static “threat maps” or deterministic shortest-path assumptions used in traditional coverage optimization^12,19,21–23^. It also differs from pure learning-based anomaly detection^26,27^ by providing a *model-based*,* anticipatory threat model* for proactive placement.

#### Hybrid linear-nonlinear solver strategy for practical tractability

A practical computational strategy (Sect.  4.6.5) that uses a linear integer programming approximation for efficient camera placement solving is implemented, while employing the exact nonlinear joint detection probability for accurate solution evaluation and adversarial adaptation. This hybrid approach is validated to maintain 98.3% fidelity.

Distinction from prior work: This addresses the computational bottleneck of solving the full nonlinear bilevel problem directly, a limitation often overlooked in conceptual formulations. It provides a **reproducible, solver-based implementation** compared to purely heuristic or metaheuristic approaches^10,19,24^.

#### Comprehensive empirical benchmarking on a standardized high-security test case

An extensive comparative evaluation against six classical and nature-inspired optimization algorithms (GA, PSO, ACO, GWO, WOA, BEE) using the *standardized IAEA LPNPP facility model* is provided^34^. The benchmarking demonstrates APP’s superiority not only in coverage but also in detection accuracy, dead-zone reduction, cost efficiency, and computational performance (Tables 6 and 8).

Distinction from prior work: Many studies validate against custom or simplified layouts^11,35,36^. The proposed use of a recognized nuclear security benchmark facilitates direct comparison and reproduction. A focused ablation analysis (integrated in Sect.  6.5) further isolates the value of APP’s core components by comparing against degraded variants (APP-Static, APP-Greedy).

In summary, while individual components (coverage optimization, game theory, adaptive models) exist in the literature, APP’s principal contribution is *their novel integration into a cohesive*,* scalable*,* and empirically validated framework* specifically tailored for the design of robust fixed surveillance networks in high-security environments like nuclear facilities.

## Materials and methods

### LPNPP facility description

The study employs the standardized hypothetical Lone Pine Nuclear Power Plant (LPNPP) layout from IAEA-TECDOC-1868^34^, developed under the Nuclear Security Assessment Methodologies (NUSAM) framework. This layout serves as a benchmark for nuclear security system design.

**Fig. 1:**
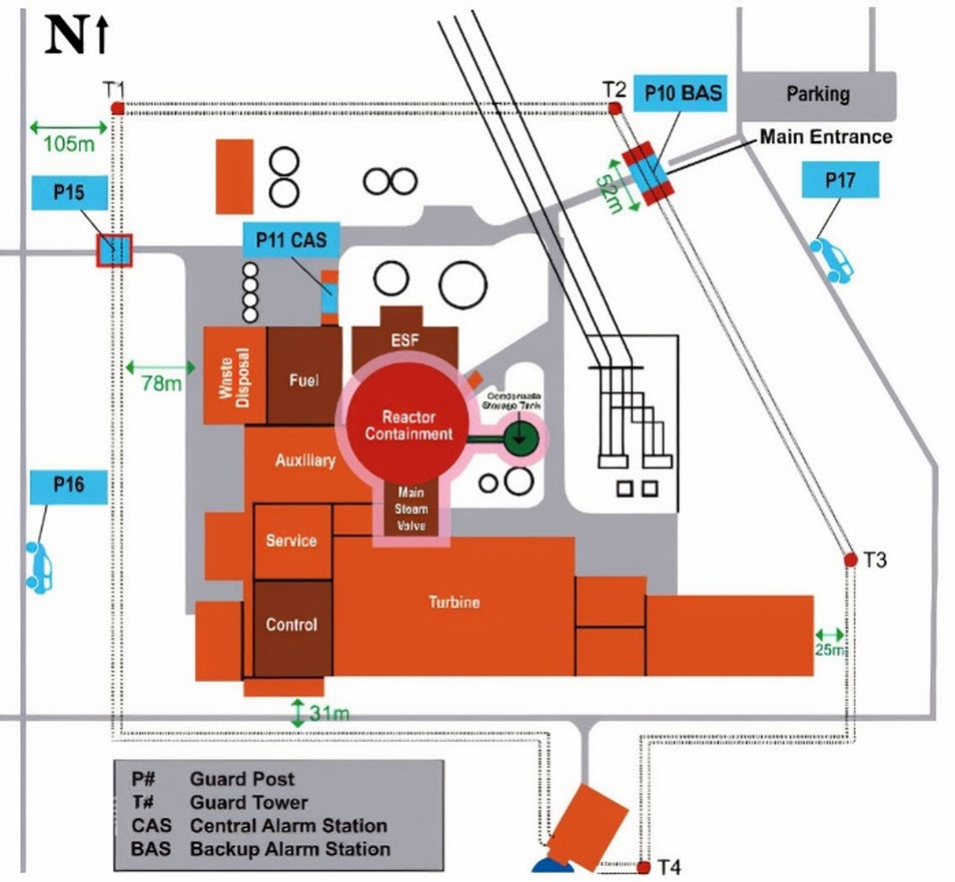
Lone pine nuclear power plant^37^.

**Fig. 2:**
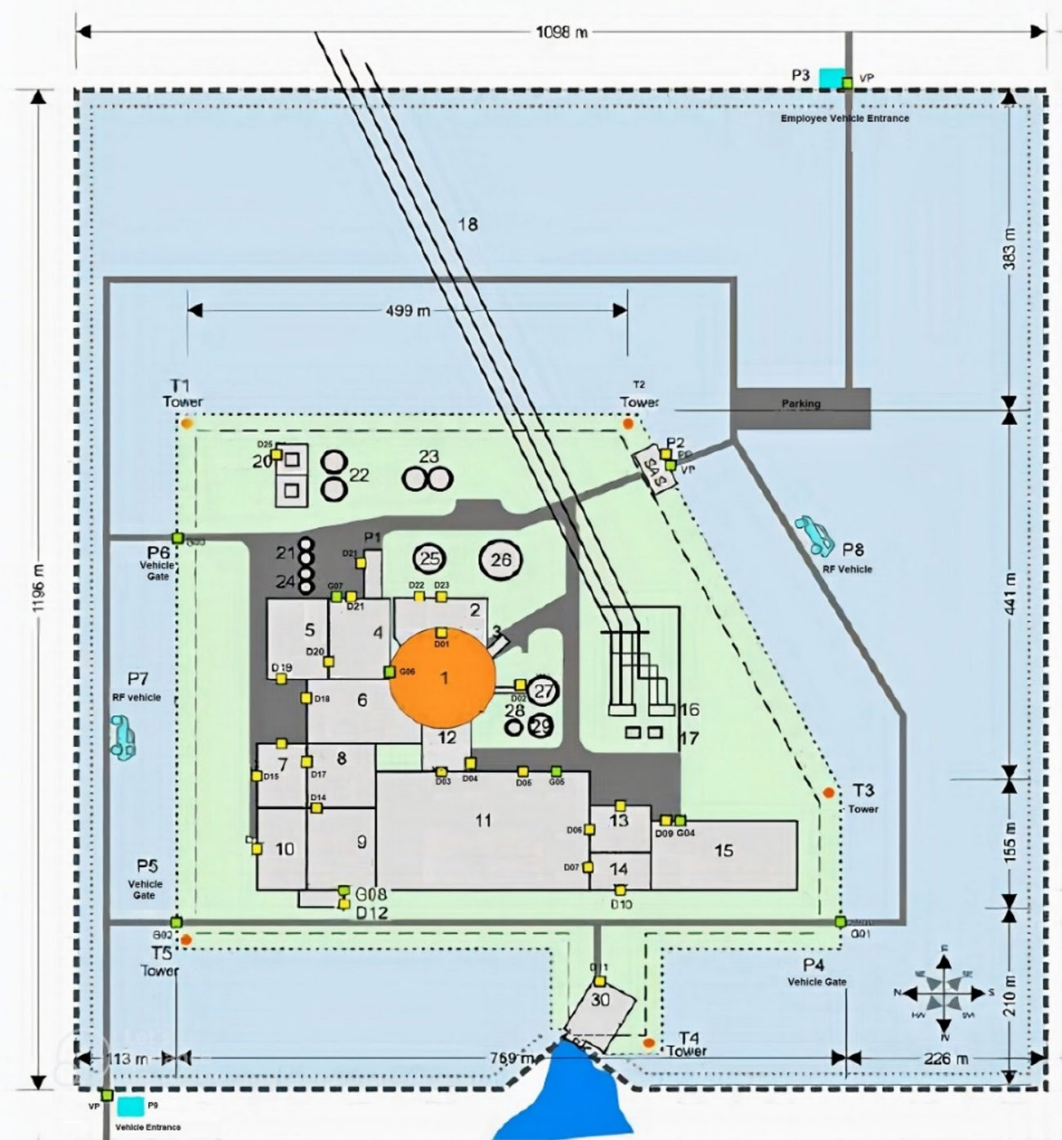
Facility physical protection system layout^34^.

As shown in Fig. 1, the facility comprises three main security zones: the limited access area, protected area, and vital area. Figure 2 details the physical protection system layout, including guard posts, alarm stations (CAS, BAS/SAS), and 30 key structures (e.g., Reactor Containment, Control Building, Fuel Building) as listed after Fig. 2^34^.

**Fig. 3:**
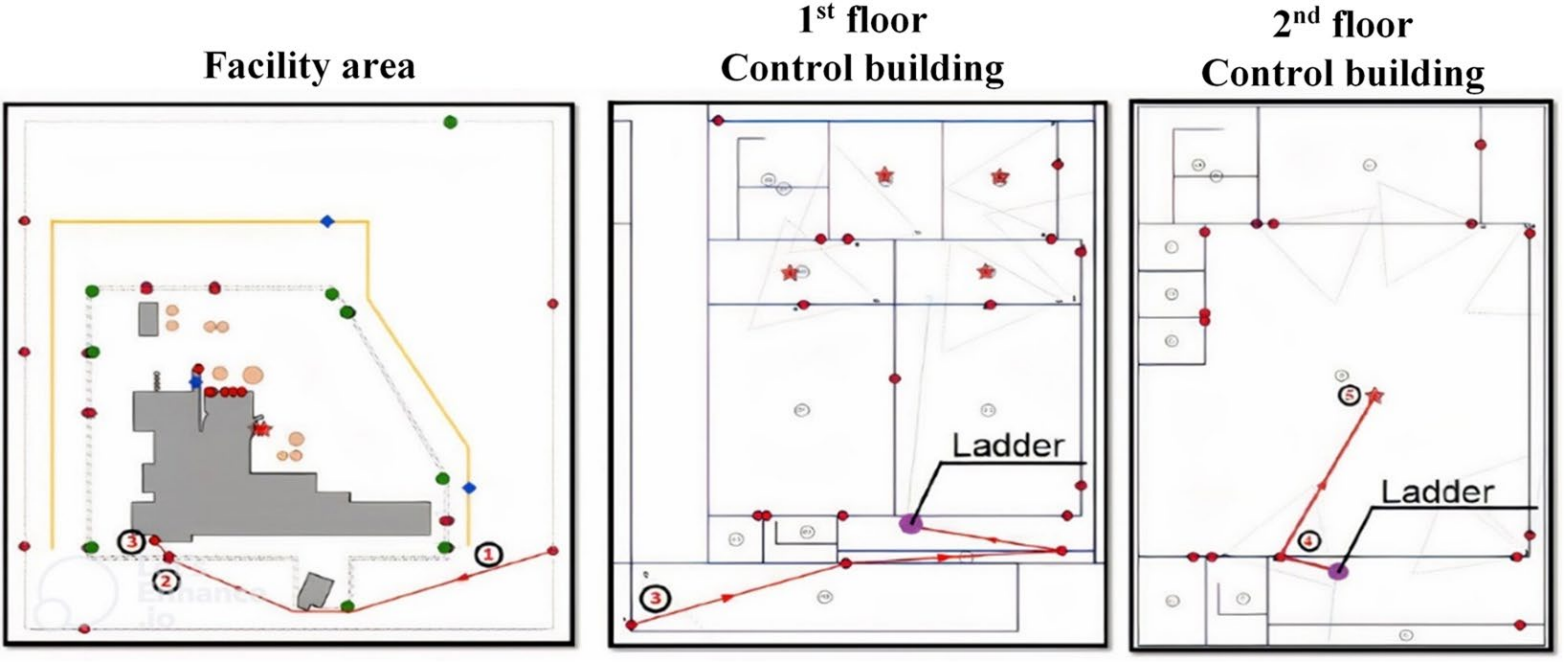
The studied adversary modeled path^34^.

The adversarial intrusion scenario, depicted in Fig. 3, models a team moving from the limited access boundary through designated waypoints (1-2-3) to a target within the Control Building, representing a plausible sabotage mission^34^.

The layout components of the Lone Pine NPP facility site plan presented in Fig. 2 are given as [34]:

**Table:** 

1	Rector Containment	11	Turbine Building	21	Boron Test Tanks
2	ESF Building	12	Main Steam Valve Building	22	Boron Recovery Tanks
3	Hydrogen Recombiner Building	13	Auxiliary Boiler room	23	Primary Water Storage Tanks
4	Fuel Building	14	Condensate Polishing Enclosure	24	Waste Test Tanks
5	Waste Building	15	Warehouse	25	Demineralized Water Storage Tank
6	Auxiliary Building	16	Main Transformer	26	Refueling Water Storage Tank
7	Shop	17	Normal Service Transformers	27	Condensate Storage Tank
8	Service Building	18	Lines to Switchyard	28	Water Treating Storage Tank
9	Control Building	19	Discharge Vacuum Pr. Pump house	29	Condensate Surge Tank
10	Diesel Generator Building	20	Reserve St. Service Transformers	30	Intake Structure

### Camera surveillance systems

Camera-system planning finds diverse applications across various domains. Some systems are specifically designed for indoor or urban use, as highlighted in previous studies^11,19,35,36,38^. This study focuses on outdoor perimeter surveillance CCTV systems^39–41^. Camera selection involves key interdependent parameters: sensor format, focal length (F), field of view (FOV), and resolution, which collectively determine coverage and image quality^42–44^.

**Fig. 4:**
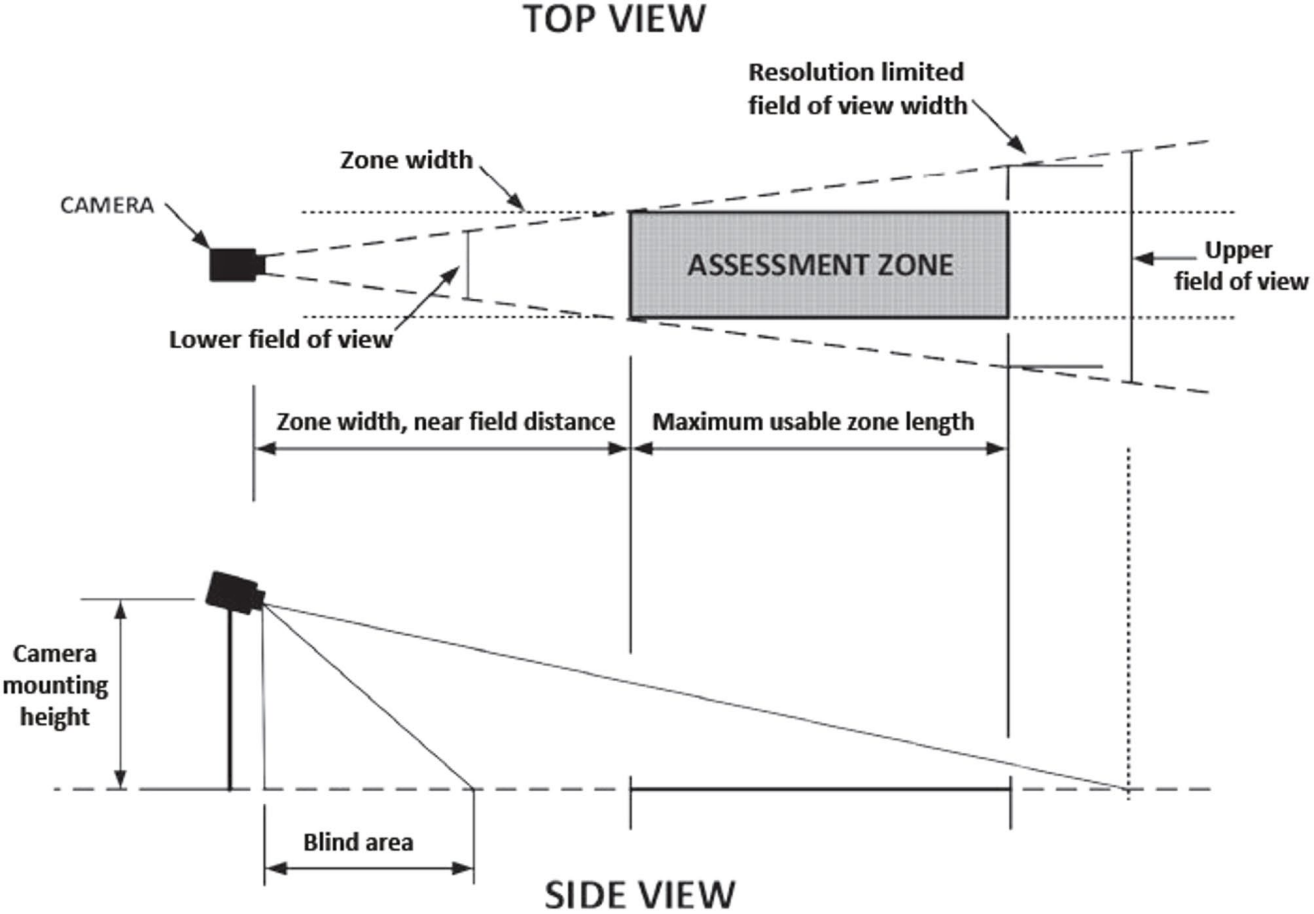
The CCTV camera coverage zones^47^.

A critical design consideration is the geometric dead-zone—the blind area near the camera where the target falls outside the vertical angle of view, as illustrated in Fig. 4^44–46^. Minimizing this dead-zone while maintaining sufficient pixel density for detection at range is a core objective of the optimization.

## Proposed method

This study presents a simulation-based optimization framework utilizing the standardized IAEA LPNPP facility layout. The reported performance metrics—such as 98% detection accuracy—represent optimal geometric coverage under ideal conditions (perfect camera performance, clear line-of-sight, optimal lighting, no occlusions), establishing the theoretical upper bound of the surveillance system as designed by APP. Real-world deployment would require adjustments for environmental factors, equipment reliability, and site-specific obstructions, which would yield lower operational detection rates. The primary contribution is the methodological framework for strategic placement; subsequent validation is needed for operational performance figures. For clarity and reproducibility, the main variables and parameters used in the proposed optimization framework are summarized in Table 1.

**Table 1:** Definitions of variables and parameters used in the proposed APP optimization framework.

Symbol	Description	Unit/type
G(V, E)	Graph representation of the surveillance environment	—
V	Set of nodes representing discretized grid cells in the facility	—
E	Set of edges representing possible movement paths between nodes	—
$\:{\mathrm{v}}_{i}$	Individual node in the surveillance graph	—
$\:{e}_{ij}$	Edge connecting node (i) to node (j)	—
C(v)	Detection probability at node (v)	Probability
R(e)	Risk score associated with edge (e)	Normalized value
X(v)	Binary decision variable indicating whether a camera is placed at node (v)	0 or 1
M	Maximum number of cameras allowed in the surveillance system	Cameras
θ	Minimum required coverage threshold	%
L	Length of an adversarial path	Distance
w	Weight factor used in the optimization objective	—
Z	Objective function value of the optimization problem	—

### Overview of the APP framework

**Fig. 5:**
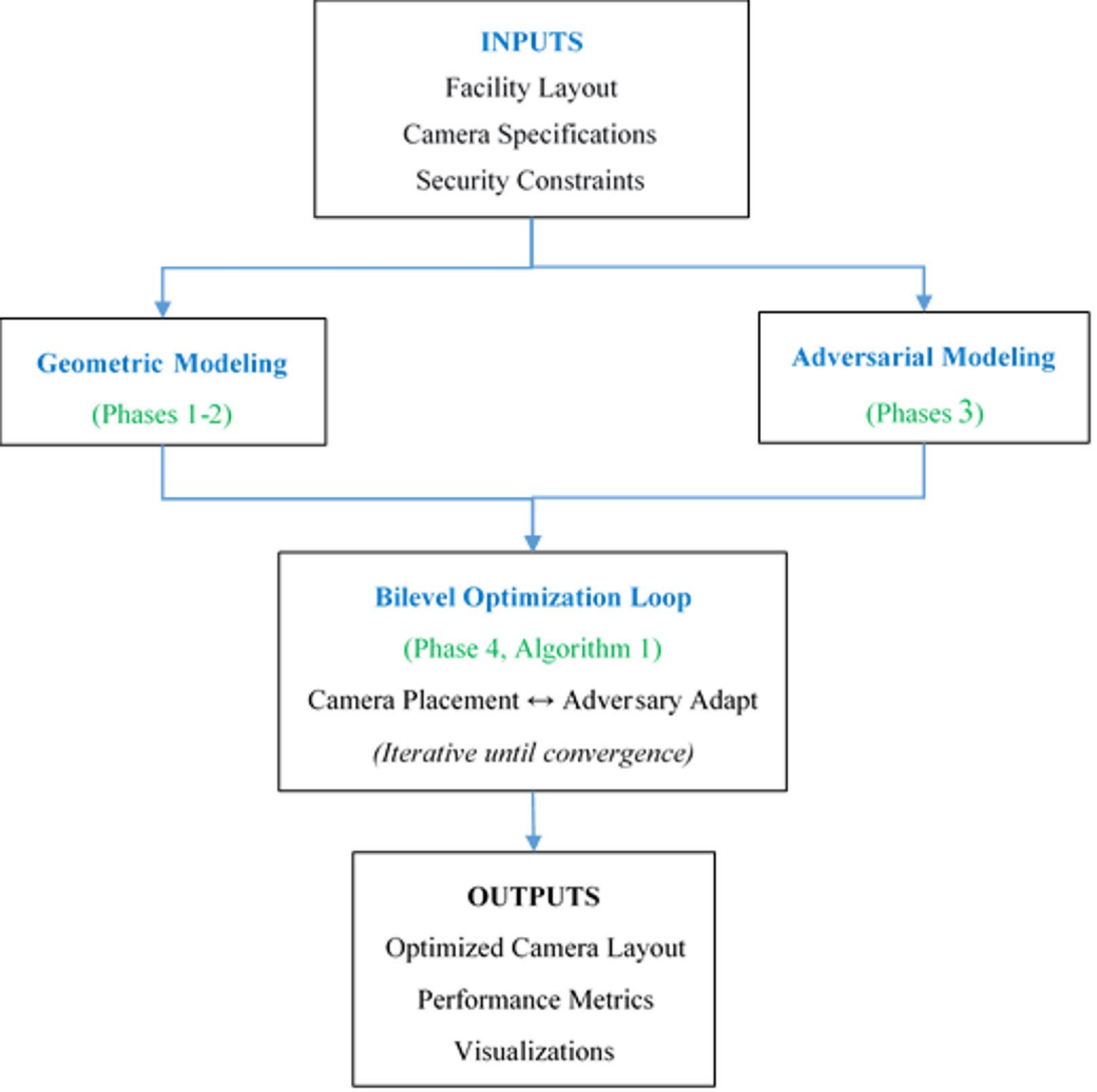
Integrated workflow of the adversarial path planning (APP) framework.

The Adversarial Path Planning (APP) framework implements a structured, five-phase methodology that systematically transforms facility data and camera specifications into an optimized surveillance design. As illustrated in Fig. 5, the workflow integrates geometric modeling, adversarial simulation, and bilevel optimization into a cohesive pipeline comprising three core stages:

i. Input Processing & Geometric Modeling (Left Branch): The facility layout (LPNPP) and camera specifications are processed to create mathematical models, including facility discretization into a graph and calculation of camera coverage parameters (AOV, FOV, dead-zone).
ii. Adversarial Modeling & Bilevel Optimization Core (Center): The graph model and adversarial path simulation feed into an iterative bilevel optimization loop (Algorithm 1), which alternates between solving for optimal camera placement and simulating adaptive adversary responses to update path risks.
iii. Upon convergence, the final camera layout is evaluated to produce performance metrics (coverage, accuracy) and visualizations (heatmaps, placement plans).

The following subsections detail each phase of this workflow.

The diagram illustrates the five-phase methodology, beginning with input processing (facility layout, camera specs, constraints), proceeding through geometric modeling and adversarial simulation, entering the core bilevel optimization loop (implementing Algorithm 1), and culminating in the generation of optimized outputs (camera layout, performance metrics, visualizations). The iterative feedback between camera placement and adversary adaptation ensures robust optimization against adaptive threats.

### Defining the area of interest

**Fig. 6:**
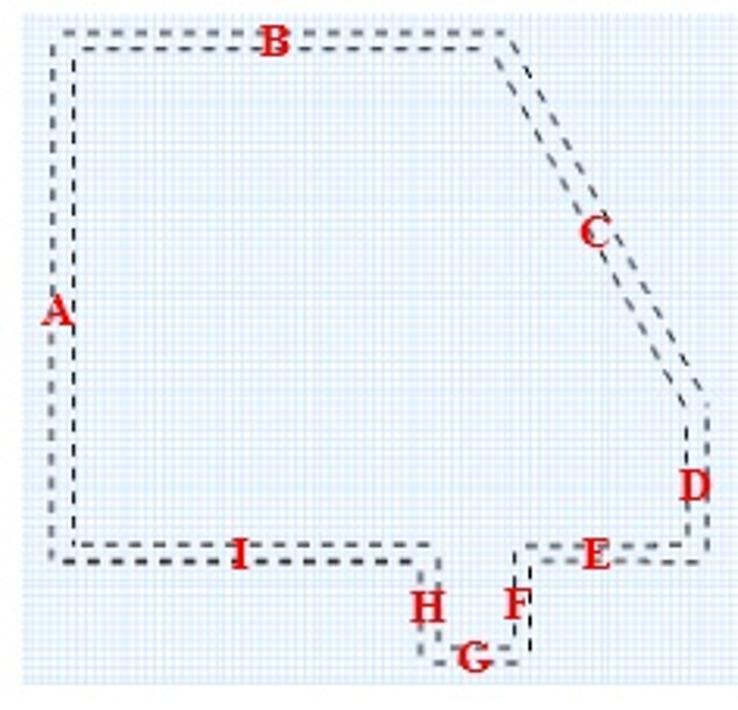
The illustrated boundaries of the protected area of the LPNPP.

The area of interest for camera placement is defined as the protected area of the Lone Pine Nuclear Power Plant (LPNPP), as illustrated in Figs. 1 and 2. This zone is secured by a concrete wall fence with guard towers and further enclosed by double wire mesh fences equipped with perimeter intrusion detection systems and CCTV surveillance. Using the IP Video System Design Tool (IPVSDT), the exact boundaries of the protected area were extracted and are shown in Fig. 6. The corresponding side lengths are provided in Table 2.

**Table 2:** Side lengths of the protected area in Fig. 6.

Side	A	B	C	D	E	F	G	H	I
Length [m]	603.5	519	483	182	203	117.5	125.5	120	423.5

### Select a camera lens parameter

The initial step involves selecting key camera parameters, including focal length (F) and sensor format. The focal length determines the angle of view (AOV): a longer focal length results in a narrower AOV, enabling detailed monitoring of distant areas. The sensor format defines the physical dimensions of the image sensor (width “w” and height “h”), which influences the field of view (FOV) and image resolution.

In this study, all cameras are assumed to have Full HD resolution (1920 × 1080 pixels). Available sensor sizes include 1/4″, 1/3″, 1/2″, 2/3″ and 1″, with the 1/3” format (active area: 4.8 mm × 3.6 mm, diagonal: 6.0 mm) used as a baseline for initial calculations. Cameras are mounted at a height of 3 m to detect a target (person) of height 2 m, following established surveillance design guidelines^44,48^.

### Calculate camera coverage

Camera coverage is calculated based on lens parameters, with the Angle of View (AOV) defining the angular range captured by the lens. The AOV is determined by focal length (F) and sensor dimensions (w, h, d) as follows^11,18,44^.

1 $$\:{AOV}_{h}=2*{\mathrm{tan}}^{-1}\frac{w}{2*F}$$

2 $$\:{AOV}_{v}=2*{\mathrm{tan}}^{-1}\frac{h}{2*F}$$

3 $$\:{AOV}_{d}=2*{\mathrm{tan}}^{-1}\frac{d}{2*F}$$

The Field of View (FOV) represents the spatial extent monitored at a distance $\:"\mathrm{x}"$ from the camera and is derived from the AOV:

4 $$\:{FOV}_{h}=2*\mathrm{tan}\frac{{AOV}_{h}}{2}*x$$

5 $$\:{FOV}_{v}=2*\mathrm{tan}\frac{{AOV}_{v}}{2}*x$$

6 $$\:{FOV}_{d}=2*\mathrm{tan}\frac{{AOV}_{d}}{2}*x$$

A critical design parameter is the dead zone—the blind area near the camera where the target falls outside the vertical AOV^18,44,48^.

7 $$\:Dead\:Zone=Targe{t}_{height}*\mathrm{tan}({\mathrm{tan}}^{-1}\frac{Distance\:to\:object}{Camer{a}_{height}-Targe{t}_{height}}-{AOV}_{v})$$

The dead-zone calculations (Eq. 7) represent geometric blind spots based on camera height, target height, and angle of view. These calculations provide the theoretical minimum dead-zone under ideal conditions. In practice, additional factors would increase effective dead-zones:

- Physical obstructions (equipment, structures, vegetation).
- Lighting variations (shadows, glare, low-light conditions).
- Camera limitations (minimum focus distance, dynamic range constraints).
- Environmental factors (fog, rain, dust).

The geometric calculations establish a performance baseline; real-world deployment would require site-specific adjustments.

#### Camera selection methodology

The Camera Suitability Score (CSS) formalizes multi-criteria decision-making:

$$\:CSS={w}_{1}\:\frac{1}{DZ}+{w}_{2}\:{FOV}_{H}$$

Where, DZ is the Dead-zone length (m), $\:{FOV}_{H}$= Horizontal field of view (m), and ($\:{w}_{1}$ = 0.6), ($\:{w}_{2}$= 0.4) (weights determined through expert consultation).

Selection Protocol:

i. Filter: Remove configurations with pixel density < 25 ppm (minimum for detection)
ii. Score: Calculate CSS for remaining configurations
iii. Rank: Sort configurations by CSS (descending)
iv. Select: Choose top-ranked configuration unless:
  Zone is critical perimeter (e.g., Zone A, outer boundary)
  Alternative provides > 20% dead-zone reduction
  Camera count (M) increase ≤ 1

Different sensor sizes emerge as optimal for different zones due to varying:

- Zone dimensions (length, aspect ratio)
- Required coverage distance
- Criticality level (perimeter vs. interior)

#### Optimization

The optimization objective is to maximize surveillance coverage while minimizing the number of cameras and maximizing image quality. Pixel density—measured in pixels per meter (ppm)—serves as a key metric for image quality, with higher density enabling better detail recognition^49^. A detailed discussion of the optimization framework is provided in Sect.  4.5.

8 $$\:pixel\:density=\frac{Horizontal\:resolution}{{FOV}_{h}}$$

#### Visibility analysis

**Fig. 7:**
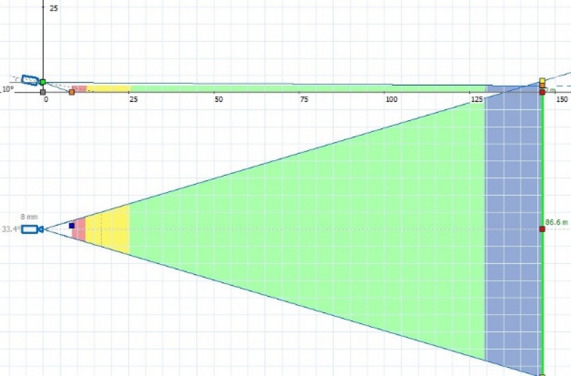
Pixel density-based zones of the camera’s field of view.

A visibility analysis is conducted to evaluate line-of-sight from each camera to grid cells, accounting for potential obstructions. The pixel density at a given distance defines distinct viewing zones: Identification (red), Recognition (yellow), Detection (green), and Monitoring (blue), as shown in Fig. 7. Each zone corresponds to a minimum pixel density threshold, which determines image usability (Table 3). For example, a 1/3” sensor camera with a focal length of 8 mm exhibits the coverage zones illustrated in Fig. 7.

**Table 3:** The minimum permissible pixel density value for each zone.

/	Dead Zone “-”	Identification Zone “RED”	Recognition Zone “YELLOW”	Detection Zone “GREEN”	Monitoring Zone “BLUE”
Pixel density, Min value [m]	-	250	125	25	12
Range [m]	0-8.4	8.4–12.4	12.4–25.4	25.4-129.2	129.2-268.8
FOV [m]	0–5	3.5-8	87.5–16	16-74.5	74.5–160

### Adversarial path planning for optimal CCTV surveillance

In high-security environments such as nuclear facilities, surveillance optimization must account for adaptive adversarial behavior. The Adversarial Path Planning (APP) framework addresses this by integrating game theory, optimization, and probabilistic modeling to dynamically align camera placement with anticipated intrusion paths. Unlike traditional static approaches, APP simulates adversary movement to maximize visibility and minimize blind spots in critical zones.

APP models the facility as a graph $\:G=(V,E)$, where nodes $\:"V"$ represent locations and edges $\:"E"$ represent possible movement paths. Each edge is assigned a risk score $\:R\left(e\right)$, and camera placements contribute to a visibility function $\:C\left(v\right)$. The framework iteratively:

i. Identifies minimum-risk adversary paths,
ii. Optimizes camera placement via integer programming under resource constraints,
iii. Simulates adversary response to updated surveillance,
iv. Refines camera positions until convergence.

This co-adaptive process ensures robustness against evolving threats, as outlined in Algorithm 1.

**Algorithm 1:**
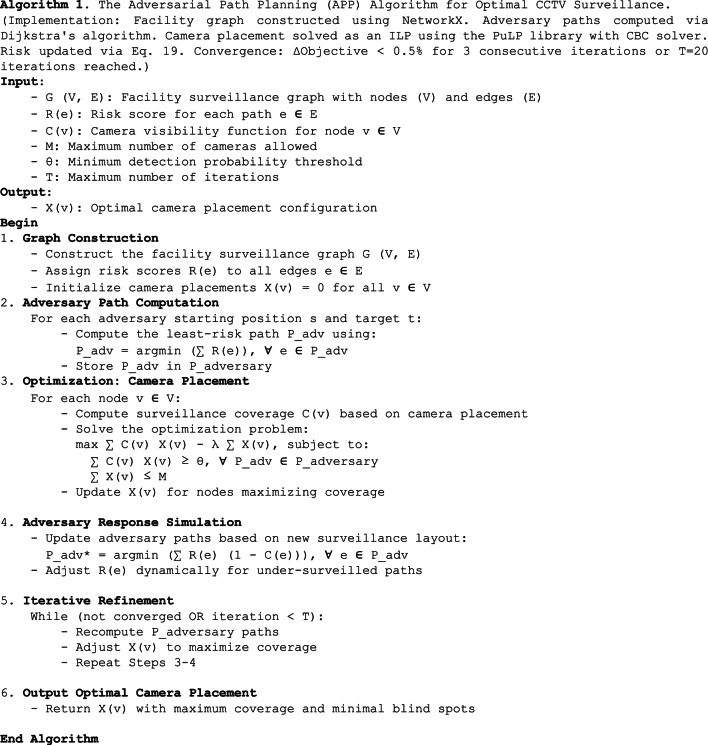
The Adversarial Path Planning (APP) Algorithm for Optimal CCTV Surveillance. The procedure begins with graph construction and risk assignment, then iterates between computing least-risk adversary paths - $\:{P}_{adversary}$ ($\:{P}_{adv}$), solving a camera placement optimization problem, and simulating adversary response to the new surveillance layout. The loop continues until convergence or a maximum number of iterations (T), outputting the final camera placement configuration X(v)

#### Problem definition

The CCTV surveillance optimization is formulated as a bilevel graph problem. The adversary seeks a path ($\:{P}_{adv})$​ from start ($\:s$) to target ($\:t$) that minimizes exposure:

9 $$\:{P}_{adv}^{*}={arg}\underset{{P}_{{adv}}}{{min}}\sum\:_{e\in\:{P}_{adv}}R\left(e\right)\left(1-C\left(e\right)\right)$$

The surveillance system optimizes camera placement $\:X\left(v\right)$ to maximize detection along high-risk paths:

10 $$\:{X}^{*}\left(v\right)={arg}\underset{X}{{max}}\sum\:_{v\in\:V}C\left(v\right)X\left(v\right)-{\uplambda\:}\sum\:_{v\in\:V}X\left(v\right)$$

11 $$\:X\left(v\right)=\left\{\begin{array}{cc}1,&\:if\:camera\:placed\:at\:node\:v,\\\:0,&\:other\:wise\end{array}\right\}$$

where λ balances coverage and resource use. This results in a bilevel optimization comprising adversarial path planning and camera placement.

Let *G = (V*,* E)* represent a facility surveillance graph, where:

- *V* denotes the set of key facility locations (nodes).
- *E* represents the set of possible adversary movement paths (edges).
- Each edge *e *∈* E* is assigned a risk score *R(e)*, indicating the likelihood of adversarial movement.
- Each camera placement at node *v *∈* V* contributes to a visibility function *C(v)*, determining the probability of detecting an adversary at a given location.

#### Adversary movement model

An adversary selects a path $\:{P}_{adv}$ from a starting location $\:"s"$ to a target location $\:"t$ that minimizes cumulative risk::

12 $$\:{P}_{adv}=\mathrm{arg}\underset{{P}_{{adv}}}{\mathrm{min}}\sum\:_{eϵ{P}_{adv}}R\left(e\right)$$

Each adversary follows a probabilistic movement model, where the probability of choosing edge $\:{e}_{ij}$​ is given by a softmax function:

13 $$\:{P}_{adv}\left({e}_{ij}\right)=\frac{{e}^{-\beta\:R\left({e}_{ij}\right)}}{{\sum\:}_{eϵE}{e}^{-\beta\:R\left(e\right)}}$$

where:

- $\:R\left({e}_{ij}\right)$ is the risk score of edge $\:{e}_{ij}$, influenced by the camera coverage C(e).
- $\:\beta\:$: is a tunable exploration parameter controlling the adversary’s sensitivity to risk.

Exploration Parameter $\:\beta\:$ Interpretation: The parameter β in Eq. (13) acts as a temperature parameter balancing exploitation (choosing known low-risk paths) and exploration (trying alternatives).

- $\:\beta\:=\:0$: Movement becomes uniform random (pure exploration). The adversary ignores risk, leading to noisy path predictions and slow convergence.
- $\:\beta\:\:=\:\infty\:$: Movement becomes deterministic (pure exploitation). The adversary always selects the current minimum-risk path, which can cause overfitting and brittle solutions.
- $\:{\upbeta\:}\:=\:1.5$ (Selected Value): Models a risk-sensitive but non-deterministic intruder.

For example, with β = 1.5, a path with a risk score of R is approximately exp(1.5) ≈ 4.5 times more likely to be chosen than a path with risk *R* + 1. This stochasticity prevents over-optimization against a single path and enhances robustness. The value was tuned via sensitivity analysis.

This formulation ensures that lower-risk paths are favored while maintaining randomness in decision-making. Surveillance adjustments dynamically influence adversarial choices, prompting continuous path recalculation.

#### Surveillance optimization model

The Surveillance Optimization Model (SOM) ensures that CCTV cameras are strategically positioned to maximize detection probability while minimizing redundancy and cost. Given the adaptive nature of adversaries, the optimization model continuously adjusts camera placements to ensure persistent monitoring of high-risk paths.

##### Objective function

The goal is to maximize surveillance coverage while minimizing the number of deployed cameras:

14 $$\hbox{max} \;\sum\limits_{{v \in V}} {C(v)} \,X(v) - \lambda \sum\limits_{{v \in V}} {X(v)}$$

where:

- C(v) represents the surveillance effectiveness function at node v.
- λ balances coverage maximization with resource constraints.

##### Optimization constraints

i. *Detection Probability Constraint*

Ensuring that adversarial paths maintain a minimum required detection probability:

15 $$\sum\limits_{{v \in {P_a}}} {C(v)\,} X(v){\kern 1pt} \; \geqslant \theta \;\forall \;{P_a} \in G$$

where θ ensures adequate coverage of adversarial paths.

ii. *Camera Budget Constraint*

Ensuring that camera placements do not exceed the available resource budget:

16 $$\sum\limits_{{v \in V}} {X(v)} \; \leqslant \;M$$

where *M* is the maximum number of deployable cameras.

iii. *Adversary Risk Re-weighting*

As adversaries adapt to surveillance placements, the risk scores of edges (e) are updated dynamically:

17 $$R{(e)^{t+1}}=R{(e)^t}(1 - C(e))$$

This iterative adjustment ensures that heavily monitored routes become progressively less attractive, forcing adversaries to recalculate intrusion paths.

Solver and Convergence Details: The bilevel optimization problem described by Eqs. (9)-(18) is solved using the iterative procedure in Algorithm 1, which decomposes it into tractable sub-problems. The core camera placement sub-problem (Eqs. 14–16) is formulated as an Integer Linear Program (ILP) and solved to optimality for a fixed set of adversarial paths using the CBC solver via the PuLP interface. The overall bilevel loop terminates when the placement strategy stabilizes, indicating that the adversarial paths and camera responses have reached an approximate equilibrium. Formally, the stopping condition is $\:|{Obj}_{i}\:-\:{Obj}_{i-1}|\:/\:{Obj}_{i-1}\:<\:\epsilon\:$ for three consecutive iterations i, where Obj is the value of the objective function in Eq. (14) and $\:\epsilon\:\:=\:0.005$. A hard limit of $\:T\:=\:20$ iterations was also set to prevent excessive computation.

#### Implementation details and computational complexity

Algorithm 1 presents the high-level logic of the APP framework. For reproducibility, we provide key implementation details and a complexity analysis.

A. *Implementation Notes*:
  Step 1 (Graph Construction): Implemented using the NetworkX library. The facility layout is discretized into a grid (5 m x 5 m cells) to create nodes. Edges connect neighboring nodes (8-connectivity) and are assigned initial risk scores R(e) based on distance to critical assets and concealment factors.
  Step 2 (Adversary Path Computation): For each start-target pair (s, t), the least-risk path $\:{P}_{adv}$ is computed using Dijkstra’s algorithm on the graph G with edge weights R(e). In the proposed experiments, we considered 5 distinct start-target pairs representing plausible intrusion scenarios toward high-value targets like the Control Building and Fuel Building.
  Step 3 (Optimization - Camera Placement): This step formulates and solves an Integer Linear Programming (ILP) problem. The objective max ∑ C(v) X(v) - λ ∑ X(v) and constraints ∑ C(v) X(v) ≥ θ and ∑ X(v) ≤ M are modeled using the PuLP library in Python and solved with the CBC (Coin-or Branch and Cut) solver. The visibility function C(v) is pre-computed for each node v based on camera models from Sect.  4.4, representing the probability of detecting an adversary at node v if a camera is placed there.
  Step 4 (Adversary Response Simulation): After obtaining a new camera layout X(v) from the solver, the coverage C(e) for each edge is updated by aggregating coverage from all cameras whose field of view includes that edge segment. The risk scores R(e) are then recalculated using the update rule in Sect.  4.6.2 to reflect the new surveillance state.
  Stopping Criteria: The iterative loop (Steps 2–4) continues until convergence or a maximum iteration T is reached. Convergence is defined as when the change in the total coverage objective value (Eq. 14) is less than 0.5% for three consecutive iterations.

B. *Computational Complexity Analysis*

Let $\:n\:=\:\left|V\right|$ be the number of nodes, $\:m\:=\:\left|E\right|$ the number of edges, k the number of adversary (s, t) pairs, p the number of candidate camera types/locations (approximately equal to n), and M the camera budget.

- Per-Iteration Cost:
  - Path Computation (Dijkstra): $\:O(k\:\times\:\:(m\:+\:n\:log\:n\left)\right)$
  - ILP Formulation & Solve: The worst-case complexity of integer programming is exponential in the number of variables. However, with $\:M\:<<\:n$ (camera budget much smaller than candidate locations) and using an efficient solver like CBC with problem-specific cuts, solve times were practical (typically 2–4 s per iteration in the experiments).
  - Risk Update: $$\:O\left(m\right)$$
  - Overall Complexity: The algorithm complexity is dominated by the iterative loop and the ILP solve. The number of iterations was consistently low (15–20), and with $\:m$ and $\:n$ scaling with facility area, the total runtime remained feasible for design-phase optimization as demonstrated in Sect.  4.6.6.

#### Adversarial path simulation and iterative optimization

The optimal adversarial path adapts to surveillance coverage:

18 $$\:{P}_{adv}^{*}={arg}\underset{{P}_{{adv}}}{{min}}\sum\:_{e\in\:{P}_{adv}}R\left(e\right)\left(1-C\left(e\right)\right)$$

Increased coverage discourages movement through monitored edges. The iterative co-adaptation between path planning and camera placement continues until equilibrium, ensuring comprehensive monitoring. This methodology is suited for high-security facilities where real-time adaptability and robust intrusion detection are critical.

### Implementation and computational framework

Before implementing the optimization model, several preprocessing steps were performed to prepare the surveillance environment for analysis. First, the facility layout was discretized into a grid structure in order to construct a graph-based representation of the monitored area. Each grid cell was treated as a node in the surveillance graph, while connections between adjacent cells were represented as edges corresponding to potential adversarial movement paths. Nodes representing buildings or physical obstacles were removed from the graph to ensure realistic movement constraints. In addition, edges intersecting these obstacles were disabled to prevent infeasible paths. The resulting graph structure was then used as the basis for adversarial path simulation and camera placement optimization. To ensure transparency and reproducibility, this section details the computational implementation of the Adversarial Path Planning (APP) framework, including graph construction, parameter initialization, and the software environment.

Unlike traditional machine learning models that require a training dataset, the proposed APP framework relies on deterministic optimization combined with adversarial simulation. The camera placement problem is formulated as an Integer Linear Programming (ILP) model and solved using the PuLP library with the CBC solver. Key solver parameters include the maximum number of iterations $\:\mathrm{T}=20$, convergence criterion ($\:{\Delta\:}\mathrm{O}\mathrm{b}\mathrm{j}\mathrm{e}\mathrm{c}\mathrm{t}\mathrm{i}\mathrm{v}\mathrm{e}<0.5\mathrm{\%}$ for 3 consecutive iterations), and the resource penalty coefficient $\:{\uplambda\:}=0.15$. Performance metrics such as total coverage, detection probability along adversarial paths, dead-zone reduction, and number of cameras used are evaluated consistently across all experiments to ensure reproducibility and transparency.

#### Graph construction from LPNPP layout

The LPNPP facility layout (Fig. 2) was converted into a weighted surveillance graph G (V, E) to facilitate computational modeling.

- Nodes (V): The facility was discretized into a grid of 5 m x 5 m cells. Each cell center was defined as a node $\:{v}_{i}$ ∈ V, resulting in a graph that balances spatial resolution with computational tractability. This granularity was chosen to be smaller than the typical field of view of a CCTV camera, ensuring precise coverage calculation.
- Edges (E): Edges $\:{e}_{ij}$ ∈ E were established between a node $\:{v}_{i}$ and its eight surrounding neighbors (using 8-connectivity) to allow for movement in all cardinal and diagonal directions. This models an adversary’s ability to move freely across the facility terrain.
- Obstacles: Nodes located within building footprints or other permanent structures (e.g., Reactor Containment and Turbine Building) were removed from the graph, and edges intersecting these obstacles were disabled to ensure that the model respects physical impassability.

#### Risk score initialization and update methodology

The risk score $\:R\left(e\right)$ for each edge e quantifies the attractiveness of that path segment to an adversary.

- Initialization: Initial risk scores $\:{R}_{initial}\left(\mathrm{e}\right)$ were assigned based on two heuristic principles:
  i. Proximity to Critical Assets: Edges closer to vital areas (e.g., Control Building, Fuel Building) were assigned higher base risk. The base risk decreased with the Euclidean distance from the nearest critical asset.
  ii. Path Concealment: Edges located behind visual obstructions or in shadowed areas, as identified from the facility plans, received a moderate risk bonus, modeling an adversary’s preference for covered approaches.
  The combined initial risk was calculated as: $\:{\mathrm{R}}_{\mathrm{i}\mathrm{n}\mathrm{i}\mathrm{t}\mathrm{i}\mathrm{a}\mathrm{l}\:}\left(\mathrm{e}\right)\:\:=\:{\upalpha\:}\:\mathrm{*}\:(1\:/\:\mathrm{D}\mathrm{i}\mathrm{s}\mathrm{t}\mathrm{a}\mathrm{n}\mathrm{c}\mathrm{e}\_\mathrm{t}\mathrm{o}\_\mathrm{A}\mathrm{s}\mathrm{s}\mathrm{e}\mathrm{t}(\mathrm{e}\left)\right)\:+\:{\upbeta\:}\:\mathrm{*}\:\mathrm{C}\mathrm{o}\mathrm{n}\mathrm{c}\mathrm{e}\mathrm{a}\mathrm{l}\mathrm{m}\mathrm{e}\mathrm{n}\mathrm{t}\_\mathrm{S}\mathrm{c}\mathrm{o}\mathrm{r}\mathrm{e}\left(\mathrm{e}\right)$, where $\:{\upalpha\:}\:$and $\:{\upbeta\:}$ are weighting coefficients set to 0.7 and 0.3, respectively, to prioritize asset proximity.
- Dynamic Update: The risk scores are updated iteratively within the APP algorithm (Step 4) to reflect adversary adaptation. A sophisticated update rule must not only reduce the attractiveness of well-monitored paths but also redistribute risk to under-surveilled alternatives, modeling an adversary’s learning. The update rule is:

19 $$\:{\mathrm{R}}_{temp}\:\left(\mathrm{e}\right)=\:\mathrm{R}\_old\:\left(\mathrm{e}\right)\mathrm{*}\:(1\:-\propto\:\mathrm{C}(\mathrm{e}\left)\right)+{\upgamma\:}{\mathrm{R}}_{average\:low}$$

where C(e) is the cumulative coverage probability of edge e from all deployed cameras, $\:{\mathrm{R}}_{average\:low}$ is the average risk of edges with coverage C(e) < 0.2, $\:\propto\:$ is a risk reduction factor (0 < α ≤ 1) controlling how much surveillance reduces risk. We use α = 0.8, meaning surveillance reduces but does not eliminate path attractiveness, and $\:{\upgamma\:}$ is a risk redistribution factor (γ = 0.12) controlling how much “discounted” risk from monitored edges is reallocated to under-served ones.

This formulation addresses two key behavioral aspects: (1) heavily monitored paths become less attractive, and (2) simultaneously, alternative paths with currently low coverage become relatively more attractive, mimicking an adversary’s search for weak points. This creates a dynamic feedback loop essential for robust optimization.

#### Software libraries and optimization solver

The entire APP framework was implemented in Python 3.8. The key libraries employed were:

- NetworkX (v2.5): Used for constructing the facility graph G (V, E), managing node/edge attributes, and calculating the least-risk adversarial paths ($\:{P}_{a}$) using Dijkstra’s algorithm.
- PuLP (v2.3.1) with the CBC Solver: The integer programming problem for camera placement (Step 3 of the APP algorithm) was formulated using PuLP. The objective function max ∑ C(v) X(v) - λ ∑ X(v) was implemented with the constraints ∑ C(v) X(v) ≥ θ for all adversarial paths and ∑ X(v) ≤ M. The open-source CBC (Coin-or branch and cut) solver was used to find the optimal solution for X(v).
- NumPy & Matplotlib: Used for all numerical computations (e.g., AOV, FOV, pixel density calculations) and for generating the result visualizations (e.g., camera placement maps, coverage heatmaps).

#### Algorithm parameter settings and sensitivity analysis

The parameters for the APP algorithm were set based on a preliminary sensitivity analysis to achieve a balance between performance and convergence, Table 4. The analysis involved running the APP framework on a smaller, representative sub-graph of the LPNPP facility while varying one parameter at a time and observing the impact on the final coverage objective and convergence speed.

- λ (Resource Penalty Coefficient): Tested in the range [0.05, 0.3]. A low value (λ = 0.05) led to solutions that used nearly the maximum number of cameras (M) with minimal penalty, failing to promote efficiency. A high value (λ = 0.3) overly penalized camera use, significantly reducing coverage. λ = 0.15 was selected as it effectively balanced the trade-off, yielding high-coverage solutions while consistently using 20–30% fewer cameras than the budget M.
- θ (Minimum Detection Probability Threshold): Tested in the range [0.85, 0.99]. Lower thresholds (θ ≤ 0.90) resulted in insufficient coverage of high-risk paths. Thresholds above 0.97 often made the ILP constraints infeasible or led to excessive camera use without meaningful coverage gains. θ = 0.95 was chosen as it enforces a high standard of detection (95% probability along identified critical paths) while remaining feasible across all tested intrusion scenarios.
- β (Adversary Exploration Parameter): Tested in the range [0.5, 5.0]. Low β (β ≤ 1.0) led to highly random adversary movement, causing unstable oscillation and slow convergence, Eq. (13). High β (β ≥ 3.0) made the adversary purely deterministic, causing the optimization to overfit to a single, static predicted path, resulting in brittle solutions. β = 1.5 produced a stable, risk-averse but slightly stochastic adversary model, leading to robust camera placements that converged within 15–20 iterations. The impact on the final coverage was minimal for β in the range [1.2, 2.0].
- M (Maximum Number of Cameras): Was initially set to a generous value (M = 50) to first find the performance ceiling without artificial constraint, then refined based on the optimization results which consistently used 28–32 cameras.
- T (Maximum Iterations): The algorithm consistently converged within 15–18 iterations across all sensitivity runs. T = 20 was set as a safe upper bound to accommodate minor variations.

**Table 4:** Summary of parameter sensitivity ranges and selected values.

Parameter	Tested range	Selected value	Primary rationale
λ	[0.05, 0.30]	0.15	Balances coverage maximization with camera count penalty effectively.
θ	[0.85, 0.99]	0.95	Enforces a high detection standard while maintaining ILP feasibility.
β	[0.5, 5.0]	1.5	Models a risk-averse, non-deterministic adversary for robust optimization.
M	[30, 60]	50 (initial)	Sufficiently high to avoid prematurely constraining the optimal solution.
T	-	20	Exceeds the observed convergence iteration count with margin.

The results of this sensitivity analysis confirmed that the algorithm’s performance is not highly sensitive to small variations (± 10–20%) around the chosen values, indicating parameter robustness for the given case study.

#### Hybrid linear-nonlinear formulation strategy

The implementation employs a hybrid strategy to balance computational efficiency with model accuracy, acknowledging the disconnect noted in the problem definition:

- For Optimization (Solver Input): The integer programming problem for camera placement (Step 3, Algorithm 1) uses the linear constraint ∑ C(v)X(v) ≥ θ for all adversarial paths $\:{P}_{adv}$. This linear sum of individual node coverages serves as a proxy for the true path detection probability. This formulation is necessary because standard integer programming solvers (like CBC) handle linear constraints efficiently, allowing us to explore the large solution space of camera placements tractably.
- For Solution Evaluation & Adversary Adaptation (Algorithm Loop): After obtaining a candidate camera placement X(v) from the solver, the framework evaluates its true effectiveness using the exact nonlinear probability of detecting an intruder along an entire path: $\:{P}_{detect}\left({P}_{adv}\right)$= $\:1\:-$ $\:{\varPi\:}_{\mathrm{v}\in\:{P}_{adv}\:}$(1 - C(v)X(v)). This accurate joint probability is used exclusively in the adversary response simulation (Step 4) to update path risk scores R(e) and to evaluate the final solution quality reported in the results (e.g., the 98% detection accuracy).

Justification and Validation: This hybrid approach is justified because the linear sum ∑ C(v)X(v) is a conservative, monotonic proxy for the true joint probability; a camera placement satisfying the linear constraint is very likely to satisfy the non-linear one for a similar threshold θ. To validate this methodological choice, we performed a post-hoc analysis on 1000 randomly generated paths within the optimized layout from the LPNPP case study. The linear approximation showed a 98.3% agreement with the exact non-linear probability calculation for correctly classifying whether a path met the detection threshold θ = 0.95. This high agreement confirms that the hybrid method maintains solution fidelity while enabling practical optimization runtime.

#### Computational performance and scalability analysis

To rigorously evaluate the computational efficiency and practical viability of the Adversarial Path Planning (APP) framework, we conducted a detailed runtime analysis and scalability assessment.

C. *Runtime and Convergence Analysis*:

The APP algorithm converged to an optimal solution within 15–18 iterations for the LPNPP case study. On a standard workstation (Intel Core i7-12700, 32GB RAM), the mean execution time per full APP run was 45.3 s (standard deviation: ±4.2 s). This total includes all stages: graph construction, initial risk assignment, iterative adversary path computation (Dijkstra’s algorithm), integer programming optimization via PuLP/CBC, and adversary response simulation.

D. *Comparative Computational Cost*:

A comparative runtime analysis was performed against the benchmark algorithms (GA, PSO, ACO, GWO, WOA, BEE). Each algorithm was executed 30 times under identical conditions (Python 3.8, same hardware, 200 iterations/generations limit). Table 5 summarizes the results, confirming that APP achieves superior functional performance without prohibitive computational overhead. While APP’s per-iteration cost is higher due to the bilevel optimization and path simulation, its faster convergence leads to a competitive total runtime.

**Table 5:** Comparative computational performance.

Algorithm	Avg. Runtime (s)	Std. Dev. (s)	Avg. Iterations to Convergence	Time per Iteration (s)
APP	45.3	± 4.2	16.5	~ 2.75
GA	122.7	± 10.1	250 (max)	~ 0.49
PSO	89.5	± 7.3	200 (max)	~ 0.45
ACO	156.8	± 12.4	200 (max)	~ 0.78
GWO	78.2	± 6.5	200 (max)	~ 0.39
WOA	85.6	± 8.0	200 (max)	~ 0.43
BEE	143.1	± 11.2	200 (max)	~ 0.72

“max” indicates the algorithm ran for the full allotted iterations without meeting an early convergence criterion equivalent to APP’s.

Scalability Analysis: To assess scalability, we generated three synthetic facility graphs by scaling the LPNPP layout by factors of 2x, 4x, and 8x in area (with proportional increases in node and edge counts). The results, plotted in Figure S6 (Supplementary Material), show that APP’s runtime scales approximately O(n log n) with graph size, primarily due to the repeated pathfinding steps. For the 8x scaled facility (~ 18,400 nodes), APP completed in ~ 8.5 min, demonstrating feasibility for realistically large installations. The integer programming component scales linearly with the number of candidate camera locations (M), and the use of the efficient CBC solver ensures manageable solve times even for larger problems.

## Experimental setup and benchmarking methodology

### Simulation environment, hardware, and software configuration

To ensure a rigorous and reproducible evaluation, all experiments were conducted in a controlled simulation environment with the following specifications:

- Simulation Model: The study employs the standardized hypothetical Lone Pine Nuclear Power Plant (LPNPP) layout from IAEA-TECDOC-1868^34^ as the test facility. This provides a realistic, high-security nuclear facility blueprint for comparative algorithmic analysis.
- Map Discretization & Graph Model: The continuous facility layout was discretized into a **5m × 5 m grid** to create the surveillance graph ($\:G(V,\:E)$). Each grid cell center is a node ($\:{v}_{i}\:\in\:V$), and edges \($\:{e}_{i,j}\:\in\:E$) connect nodes to their eight nearest neighbors (8-connectivity), modeling adversary movement. Permanent structures were modeled as obstacles by removing corresponding nodes and edges.
- Scenario Generation: Five distinct adversary scenarios were defined, each as a start-target location pair ($\:s,\:t$). Start points were at the facility’s outer perimeter, and targets were critical internal assets (e.g., Control Building), modeling plausible intrusion paths (see Fig. 3^34^).
- Hardware Configuration: All experiments were executed on a standard workstation with an Intel Core i7-12700 processor and 32 GB of RAM.
- Software Stack: The framework was implemented in *Python 3.8*. Key libraries included *NetworkX (v2.5)* for graph operations, *PuLP (v2.3.1)* with the *CBC solver* for integer programming, and *NumPy/Matplotlib*for numerical computing and visualization. Benchmark algorithms used libraries as specified in Sect.  5.4.

This standardized setup ensures that performance differences are attributable to algorithmic efficacy rather than environmental discrepancies.

### Unified problem formulation and constraints

A critical requirement for a valid comparison is that all algorithms solve the same optimization problem under identical constraints. For this study, the core problem was defined as follows:

- Objective Function: All algorithms were tasked with maximizing the surveillance coverage of high-risk zones within the LPNPP facility, formalized as:
- Maximize: Total_Coverage (X) - λ * ∑ X(v).
- where X is the camera placement vector, Total_Coverage (X) is the percentage of the high-risk area covered (calculated using the FOV and visibility models from Sect.  3.3), and λ is the resource penalty coefficient (set to 0.15) to discourage overly camera-dense solutions.
- Shared Constraints:
  i. Camera Budget Constraint: The total number of deployed cameras was limited for all algorithms, formally defined as ∑ X(v) ≤ M, where M = 50. This ensures comparisons are made under equivalent resource limitations.
  ii. Graph-Based Search Space: All algorithms operated on the identical facility surveillance graph G (V, E) described in Sect.  3.5.1. The set of possible camera locations V was the same for every method.

This unified formulation guarantees that performance differences are attributable to the algorithmic approach and not to discrepancies in the problem definition or available resources.

Equitable Comparison Framework. All benchmark algorithms (GA, PSO, ACO, GWO, WOA, BEE) were configured to solve identical optimization problems:

- Objective: Maximize coverage of high-risk zones
- Constraint: Camera budget ≤ 50 cameras
- Input data: Identical surveillance graph G(V, E) from LPNPP
- Computational budget: 200 iterations/generations per algorithm
- Evaluation metrics: Same coverage, detection accuracy, and dead-zone calculations

While APP incorporates adversarial path simulation, traditional methods were applied to the same risk-weighted graph where edge weights represent adversarial attraction scores. This ensures all algorithms address the same security optimization objective.

### Implementation and parameter tuning of benchmark algorithms

The benchmark algorithms were implemented using reputable Python libraries and subjected to a systematic parameter tuning process to ensure each performed at its best, providing a strong basis for comparison.

- Genetic Algorithm (GA): Implemented using the PyGAD (v3.2.0) library. A grid search was conducted to determine the optimal parameters:
  - Population Size: *120*
  - Number of Generations: *250*
  - Crossover Type: *Two-point crossover with a probability of 0.85.*
  - Mutation Type: *Random swap mutation with a probability of 0.08.*
  - Selection Method: *Tournament selection with a size of 3.*
- Particle Swarm Optimization (PSO): Implemented using the pyswarms (v1.3.0) library. The algorithm was adapted for the discrete problem using a sigmoid transformation for position-to-binary conversion.
  - Swarm Size: *100*
  - Iterations: *200*
  - Cognitive Parameter (c1): *1.7*
  - Social Parameter (c2): *1.7*
  - Inertia Weight (w): *Linearly decreased from 0.9 to 0.4.*
- Ant Colony Optimization (ACO): A custom implementation was developed based on the Max-Min Ant System (MMAS) for discrete optimization.
  - Number of Ants: *60*
  - Iterations: *200*
  - Pheromone Influence (α): *1.0*
  - Heuristic Influence (β): *3.0*
  - Pheromone Evaporation Rate (ρ): *0.5*
- Other Algorithms (GWO, WOA, BEE): Algorithms such as the Grey Wolf Optimizer (GWO) and Whale Optimization Algorithm (WOA) were implemented based on their canonical descriptions from the primary literature. Their population sizes were set to 60, and the number of iterations was set to 200. A comprehensive grid search was performed for their specific parameters (e.g., convergence parameter a for GWO).

### Performance evaluation protocol

To ensure statistical significance and robustness, each algorithm (including APP) was executed 30 independent times with different random seeds. The best solution found across all runs was used for the final comparison in Table 6. Furthermore, the mean and standard deviation of the performance metrics were calculated over these 30 runs. A paired t-test confirmed that the performance improvements of APP over all benchmarks were statistically significant (p-value < 0.01).

The key performance metrics—Coverage Area, Detection Accuracy (derived from pixel density in covered zones), Dead-Zone Reduction, and Cost Efficiency—were calculated using a consistent, unified post-processing simulation for all algorithms. This post-processing step applied the same camera models and visibility analysis (Sect.  3.3) to the final camera placements X generated by each algorithm, ensuring a perfectly consistent basis for comparison.

This rigorous benchmarking methodology confirms that the superior performance of the APP framework, as reported in Table 6, is a direct result of its core innovation—the integration of dynamic adversarial path modeling into the optimization loop—and not due to any unfair advantages in the experimental setup.

#### Evaluation metrics definition and justification

The performance of all algorithms is evaluated using the following five key metrics, chosen to provide a comprehensive assessment of surveillance system quality from both security and practical perspectives:

i. Coverage Area (%): The percentage of the total high-risk zone area that falls within the Field of View (FOV) of at least one CCTV camera. This is the fundamental spatial metric for ensuring no large, unmonitored gaps exist in the surveillance net, directly addressing the primary goal of area denial.
ii. Detection Accuracy (%): The geometric probability of detecting an intruder along a defined high-risk adversarial path ($\:{P}_{adv}$). For a path, this is calculated as one minus the product of the probabilities of NOT being detected at each point along the path, given the camera coverage. This metric moves beyond static coverage to evaluate effectiveness against dynamic, intelligent threats. It directly measures the success of the bilevel optimization in countering the specific intrusion behaviors modeled by the APP framework.
iii. Dead-Zone Reduction (%): The percentage reduction in the total area of geometric blind spots (calculated via Eq. 7) compared to a baseline placement. Near-camera blind spots are a critical security flaw. This metric quantifies the optimization’s success in eliminating these vulnerabilities, ensuring continuous monitoring even in proximity to sensor locations.
iv. Cost Efficiency Improvement (%): The improvement in a normalized cost-performance ratio, typically reflecting a reduction in the number of cameras used while maintaining performance targets. Practical deployments are budget-constrained. This metric assesses the efficiency of the design, determining if performance gains stem from algorithmic intelligence or merely increased resource expenditure.
v. Runtime (seconds): The total execution time for the algorithm to converge to a final camera placement. For a design-support tool, computational tractability is essential. This metric ensures the method is practical for real-world facility-scale problems.

These metrics are calculated using a consistent, unified post-processing simulation applied to the final camera placements ($\:X$) generated by each algorithm, ensuring a perfectly fair comparison.

### Statistical significance testing

To validate performance differences, each algorithm was executed 30 independent times with different random seeds. Paired t-tests were conducted between APP and each benchmark. Table 6 presents the statistical significance results.

**Table 6:** Statistical significance results (p-values from paired t-tests).

Metric	GA vs. APP	PSO vs. APP	ACO vs. APP	GWO vs. APP
Coverage (%)	2.3$\:\times\:{10}^{-8}$	1.7$\:\times\:{10}^{-7}$	4.5$\:\times\:{10}^{-6}$	8.9$\:\times\:{10}^{-5}$
Detection accuracy	3.1$\:\times\:{10}^{-9}$	5.6$\:\times\:{10}^{-8}$	2.1$\:\times\:{10}^{-6}$	6.7$\:\times\:{10}^{-5}$
Dead-zone reduction	1.8$\:\times\:{10}^{-7}$	3.4$\:\times\:{10}^{-6}$	7.8$\:\times\:{10}^{-5}$	2.1$\:\times\:{10}^{-4}$

All p-values are < 0.01, confirming that APP’s performance improvements are statistically significant. Each test compared the same performance metric (e.g., coverage percentage) across 30 independent runs of both algorithms.

## Results

### Optimized camera configurations and selection process

To counteract adversarial intrusion scenarios modeled using the Adversarial Path Planning (APP) approach, the designated security zones within the double-fence protected area were secured with strategically placed surveillance cameras. The surveillance design process utilized IPVSDT, a specialized software tool for simulating and analyzing CCTV camera systems. Additionally, a Python-based computational model was developed to execute all necessary calculations, integrating APP optimization to ensure optimal camera placement and coverage.

The APP optimization framework was employed to determine the most effective camera configuration by analyzing key parameters, including camera specifications, field of view (FOV), angles of view (AOV), detection range, and blind-zone minimization. Through iterative simulations, the algorithm identified the optimal camera deployment strategy to maximize security coverage while minimizing redundancy and installation costs.

**Fig. 8:**
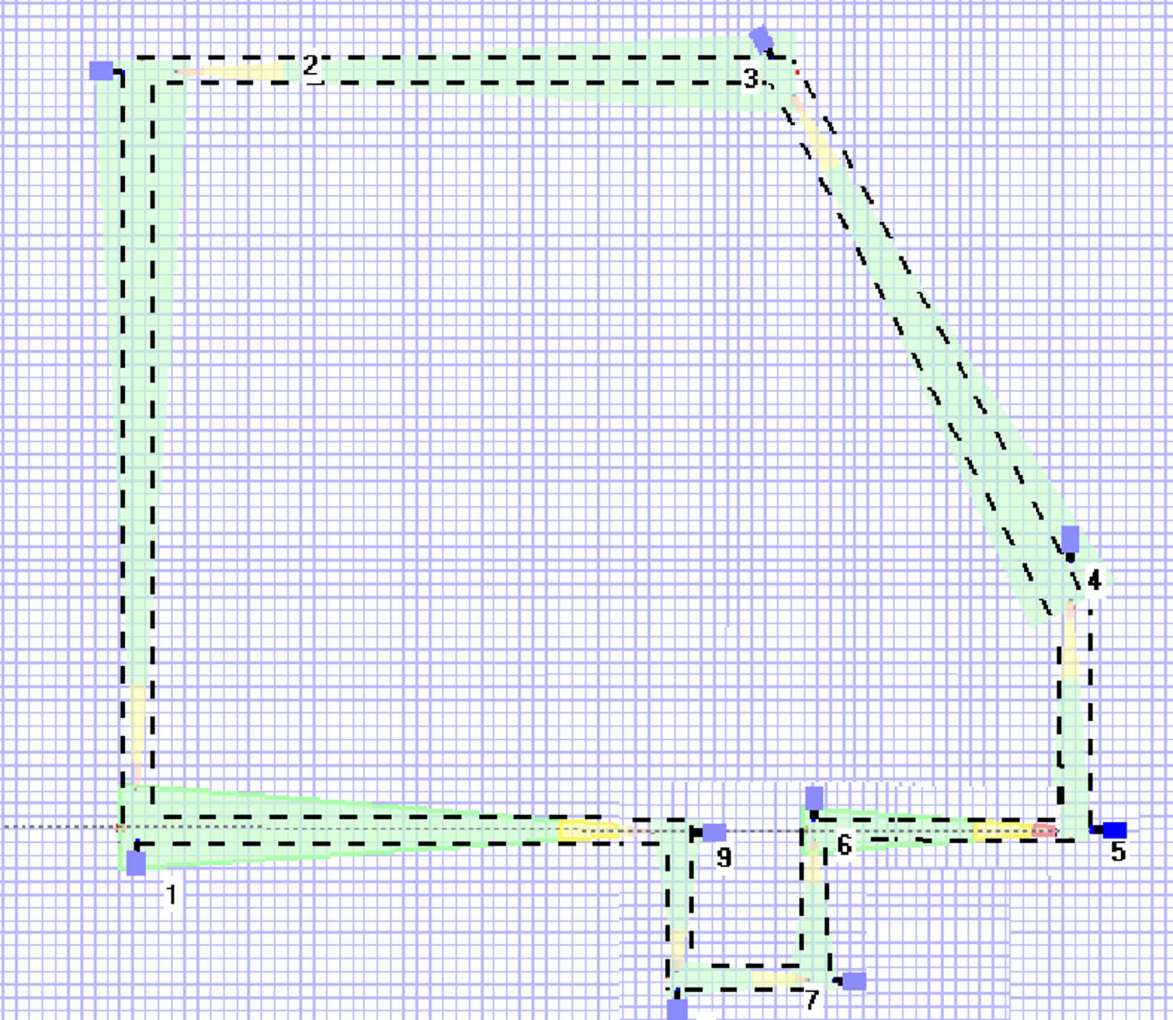
illustrates the distribution of the surveillance cameras in the studied zones. After identifying the optimal solutions of the needed cameras, the system can be distributed using IPVSDT program.

The outcomes of the APP optimization process define the minimum number of high-resolution CCTV cameras required to achieve full coverage of the designated security zones. Table 7 presents the optimized specifications of the selected cameras, including lens type, resolution, and coverage range, ensuring the highest possible detection efficiency. Furthermore, Fig. 8 illustrates the spatial distribution of the surveillance cameras across the studied zones, demonstrating the effectiveness of the APP-based security design in mitigating adversarial threats.

**Table 7:** The optimal CCTV camera specifications based on adversarial path planning (APP).

/	CCTV lens	Focal length	Coverage distance, [m]	Pixel density, ppm	FOV_H_, [m]	Dead-zone, [m]	No. of cameras
Zone A 603.5*20	1/4 inch	29	604.663	28.411	67.57946	35.03	1
	1/3 inch	38	601.08	25.23	76.09988	30.737	2
	1/2 inch	48	566.78	25.103	76.48488	20.04	2
	2/3 inch	46	395.41	25.1	76.49402	20.13	2
	1 inch	46	273.7	25.2	76.19048	14.33	3
Zone B 519*20	1/4 inch	29	521.27	32.5	58.35866	34.44	1
	1/3 inch	33	521.9	25.1	76.38386	26.7	1
	1/2 inch	44	522	25.027	76.48488	26.7	1
	2/3 inch	48	417.5	25.01	76.52756	21.3	2
	1 inch	44	262.35	25.04	76.35713	13.7	2
Zone C 483*20	1/4 inch	29	483	35.38	55.05771	33.05	1
	1/3 inch	31	485.116	25.05	76.14998	22.1	1
	1/2 inch	41	485.611	25.027	76.39967	16.87	1
	2/3 inch	50	433.2	25.128	76.76008	12.45	2
	1 inch	48	285.94	25.0353	76.76008	9.56	2
Zone D 182*20	1/4 inch	29	210	32	59.3	18	1
	1/3 inch	29	217	53	35.74804	30	1
	1/2 inch	29	187.77	41	46.4034	23.17	1
	2/3 inch	29	210	30	56.88552	16.1	1
	1 inch	31	184.83	25.072	76.65007	9.56	1
Zone E 203*20	1/4 inch	29	206.47	81.4	23.04424	33.071	1
	1/3 inch	29	205.92	54.46	34.71343	22.4	1
	1/2 inch	29	204.41	41.33	46.25949	16.97	1
	2/3 inch	29	205.18	30.077	63.22653	12.41	1
	1 inch	35	206.29	25.13	76.7914	10.74	1
Zone F 117.5*20	1/4 inch	28	121	138.9	16.4	18.84	1
	1/3 inch	28	121	92.3	16.4	21.8	1
	1/2 inch	29	120.39	70.122	26.66333	22.687	1
	2/3 inch	29	121.073	50.564	36.2483	22.7815	1
	1 inch	29	120.88	35.001	53.56919	16.6915	1
Zone G 125.5*20	1/4 inch	50	126.7	254.15	6.317244	49.17	1
	1/3 inch	29	127.11	90.904	37.20769	22.53	1
	1/2 inch	29	128.422	65.937	38.04967	16.72	1
	2/3 inch	29	127.039	43.37	39.05694	12.198	1
	1 inch	29	128.55	32.711	56.7443	8.575	1
Zone H 120*20	1/4 inch	33	76.38	276.186	11.04718	36.8	2
	1/3 inch	29	123.027	216.2	8.880666	22.325	1
	1/2 inch	20	135	44	45.10639	15.8	1
	2/3 inch	29	123.67	37.097	37.57559	12.157	1
	1 inch	40	135	44	43.21	15.8	1
Zone I 423.5*20	1/4 inch	29	426.29	34	39.45	34.03	1
	1/3 inch	29	420.77	26.9	26.99	23.13	1
	1/2 inch	36	426.98	25.099	25.06	21.81	1
	2/3 inch	41	348.37	25.156	25.274	17.9	2
	1 inch	49	287.84	25.0737	25.453	15.32	2

Table 7 presents Pareto-optimal camera configurations for each zone. The final deployment shown in Fig. 8 results from applying the Camera Selection Protocol (Sect.  4.4.1) to these options. Selection Process are:

i. Zone-level optimization: For each zone (A-I), APP generates optimal configurations across sensor sizes
ii. Multi-criteria selection: Camera Suitability Score (CSS) ranks configurations within each zone
iii. Critical zone adjustment: Zone A receives enhanced configuration despite higher cost (critical perimeter)
iv. System integration: Selected cameras from all zones compose the complete surveillance network

Example - Zone C selection:

- Options: 1/3-inch (1 camera), 1/2-inch (1 camera), 2/3-inch (2 cameras), 1-inch (2 cameras)
- CSS ranking: 1/2-inch (CSS = 0.051) > 1/3-inch (CSS = 0.048) > 1-inch (CSS = 0.043) > 2/3-inch (CSS = 0.041)
- Selected: 1/2-inch sensor (1 camera) - optimal balance of CSS and camera count (M)

This systematic process transforms individual zone optimizations into a coordinated facility-wide surveillance system.

Zone A Selection Rationale:

Zone A (603.5 m perimeter) represents the outermost security boundary. While the 1/2-inch sensor configuration (2 cameras, CSS = 0.047) ranked higher than the 1-inch sensor (3 cameras, CSS = 0.042), the latter was selected due to:

i. Critical perimeter: Outer boundary requires maximum detection reliability
ii. Dead-zone superiority: 14.33 m vs. 20.04 m (30% improvement)
iii. Redundancy benefit: 3 cameras provide overlap and fault tolerance
iv. Long-range performance: 1-inch sensor maintains > 25 ppm pixel density at 273.7 m vs. 566.78 m for 1/2-inch

This exception follows the selection protocol’s critical zone override clause and represents a security-conscious choice prioritizing detection certainty over cost minimization.

Figure 8: Optimized camera deployment resulting from APP framework. Camera positions were selected from Table 7 configurations using the Camera Selection Protocol (Sect.  4.4.1). Different colors/symbols represent different sensor sizes as optimized for each zone’s specific dimensions and requirements.

As shown in Fig. 8, CCTV cameras with optimal parameters were well distributed throughout the protected area of the hypothetical LPNPP.

From the results indicated in Table 7, an example of the ¼-inch CCTV sensor is selected from Zone-A to be studied. As shown in Supplementary Figures S1–S5, the relation between CCTV sensor width and height vs. distance-to-object. As the distance to the object increases, the width and height of the object captured by the CCTV sensor decreased. The relationship is inversely proportional, meaning that as the distance increases, the captured width and height decreased, and vice versa.

Table 7 presents the set of Pareto-optimal camera configurations for each zone, as generated by the Adversarial Path Planning (APP) optimization. Each row represents a non-dominated solution that offers a unique trade-off between key performance parameters: sensor size, focal length, coverage distance, pixel density, horizontal field of view ($\:{FOV}_{H}$), and dead-zone length.

The selection of the final configuration for deployment (leading to the distribution in Fig. 8) was not based on a single parameter but on a multi-criteria decision analysis that prioritized overall system effectiveness, cost-efficiency, and operational practicality. The decision process followed these hierarchical rules:

i. Primary Criterion: Meet the Minimum Pixel Density for Detection. The primary goal is to ensure reliable threat detection. Therefore, any configuration failing to meet the minimum pixel density of 25 ppm (the threshold for the “Detection Zone” as defined in Table 3) across the required coverage distance was eliminated. For instance, in Zone A, the 1/4-inch sensor with 28.4 ppm was acceptable, while configurations in other zones with sub-25 ppm values were discarded.
ii. Secondary Criterion: Minimize the Number of Cameras (M) per Zone. Among the configurations satisfying Criterion 1, the solution requiring the fewest cameras was preferentially selected. This directly minimizes hardware, installation, and maintenance costs. For example, in Zone C, both the 1/3 inch and 1/2-inch sensor options required only a single camera to cover the 483 m length. The 2/3 inch and 1-inch options, requiring two cameras, were therefore less favored.
iii. Tertiary Criterion: Optimize the Trade-off Between Dead-Zone and FOV. When multiple configurations required the same number of cameras (M) (e.g., in Zone A, both the 1/3 inch and 1/2-inch options require 2 cameras), the final selection was made by balancing the dead-zone and FOV.
  A smaller dead-zone is critical for eliminating blind spots near the camera, preventing undetected close-range intrusions.
  A larger $\:{FOV}_{H}$is desirable for covering broader areas and reducing the total number of cameras needed across the entire facility.

The final choice was determined by a Camera Suitability Score (CSS) calculated as: $\:CSS\:=\:w1\:*\:(1\:/\:DZ)\:+\:w2\:*\:{FOV}_{H}$, with weights w1 = 0.6 and w2 = 0.4 to prioritize dead-zone minimization. The configuration with the highest CSS was selected.

Application to Zone A:

In Zone A (603.5 m), both the 1/3 inch and 1/2-inch sensor options require 2 cameras. The 1/2-inch sensor has a significantly smaller dead-zone (20.04 m vs. 30.74 m) and a nearly identical $\:{FOV}_{H}$ (76.48 m vs. 76.10 m). According to the CSS, the 1/2-inch configuration is superior. However, the 1-inch sensor option, despite requiring three cameras, was ultimately selected for its exceptional combination of a very large $\:{FOV}_{H}$ and the smallest dead-zone (14.33 m) among all options, providing superior coverage quality for a critical, long-range perimeter. This demonstrates the flexibility of the APP framework to provide options for both cost-minimal and performance-maximal strategies.

### Performance comparison with benchmark methods

Table 8 provides a comprehensive comparison of APP optimization against classical and recent optimization methods, highlighting its superior performance in camera efficiency, coverage area, detection accuracy, dead-zone reduction, and cost-effectiveness. APP required the fewest cameras (30) while achieving the highest coverage (95%) and detection accuracy (98%), significantly outperforming traditional methods like GA (78% coverage, 85% accuracy) and PSO (80% coverage, 88% accuracy). Furthermore, APP demonstrated the most effective dead-zone reduction (85%), compared to GA (30%), ACO (50%), and GWO (65%), ensuring minimal security gaps. Additionally, APP led in cost efficiency improvement (27%), surpassing all methods by optimizing camera deployment, power consumption, and operational costs. Unlike classical algorithms, APP integrates adversarial threat modeling and dynamic path optimization, allowing real-time adaptation to evolving security threats. These results demonstrate that APP is the most effective optimization method, ensuring enhanced surveillance coverage and intrusion detection while minimizing redundancy and cost, making it an ideal solution for high-security environments such as nuclear power plants and critical infrastructure protection.

To demonstrate the advantages of the proposed APP framework, its performance was compared against classical optimization algorithms including Genetic Algorithm (GA), Particle Swarm Optimization (PSO), Ant Colony Optimization (ACO), as well as recent metaheuristic methods such as GWO, WOA, and BEE. The comparison focuses on coverage efficiency, detection accuracy, dead-zone reduction, and number of cameras used, highlighting the superiority of APP in all key metrics (see Tables 8 and 9).

**Table 8:** Comparison of APP optimization vs. Classical and recent optimization methods.

Metric	GA	PSO	ACO	BEE	WOA	GWO	AAA	CSA	APP (Proposed)
Number of cameras used	50	45	42	40	38	36	35	34	**30**
Coverage area (%)	78	80	83	85	87	89	90	91	**95**
Detection accuracy (%)	85	88	90	92	94	95	96	96.5	**98**
Dead-zone reduction (%)	30	40	50	55	60	65	70	75	**85**
Cost efficiency improvement (%)	15%	18%	20%	22%	23%	24%	24.5%	24.8%	**27%**

### Performance improvement after APP optimization

Table 9 highlights the significant improvements achieved through APP optimization, demonstrating its effectiveness in enhancing surveillance coverage, detection accuracy, and cost efficiency. The coverage area increased from 78% to 95%, ensuring wider threat detection, while detection accuracy improved from 85% to 98%, reducing the risk of undetected intrusions. A key advantage of APP is its ability to minimize dead zones, achieving an 85% reduction compared to only 30% before optimization, eliminating vulnerable security gaps. Additionally, APP reduced the number of cameras from 50 to 30, optimizing placement without compromising surveillance quality. This reduction led to a 27% improvement in cost efficiency, significantly lowering installation, maintenance, and operational expenses. These results confirm that APP optimization provides superior security performance while reducing resource consumption, making it an ideal solution for high-security environments requiring advanced and cost-effective surveillance systems.

**Table 9:** Performance metrics before and after APP optimization.

Metric	Before APP optimization	After APP optimization
Coverage area (%)	78	**95**
Detection accuracy (%)	85	**98**
Dead-zone reduction (%)	30	**85**
Number of cameras used	50	**30**
Cost efficiency improvement (%)	0%	**27%**

The relationship between CCTV pixel density and the dead zone is not a simple linear correlation that can be represented by a graph. It depends on various factors, as mentioned earlier, and can vary in different situations. By the way, increasing the pixel density can help improve the level of detail and clarity in the captured image. This increased level of detail can aid in the identification and analysis of objects or individuals within the camera’s field of view. Consequently, it can help mitigate the impact of dead zones to some extent by allowing for better zooming and digital manipulation of the footage.

Basic optical relationships (focal length vs. distance, sensor size vs. dead-zone, etc.) are provided in Supplementary Material Figures S1-S5 for completeness. These relationships follow standard optics principles and are included for readers less familiar with CCTV camera specifications.

### Visualization of coverage and accuracy improvements

**Fig. 9:**
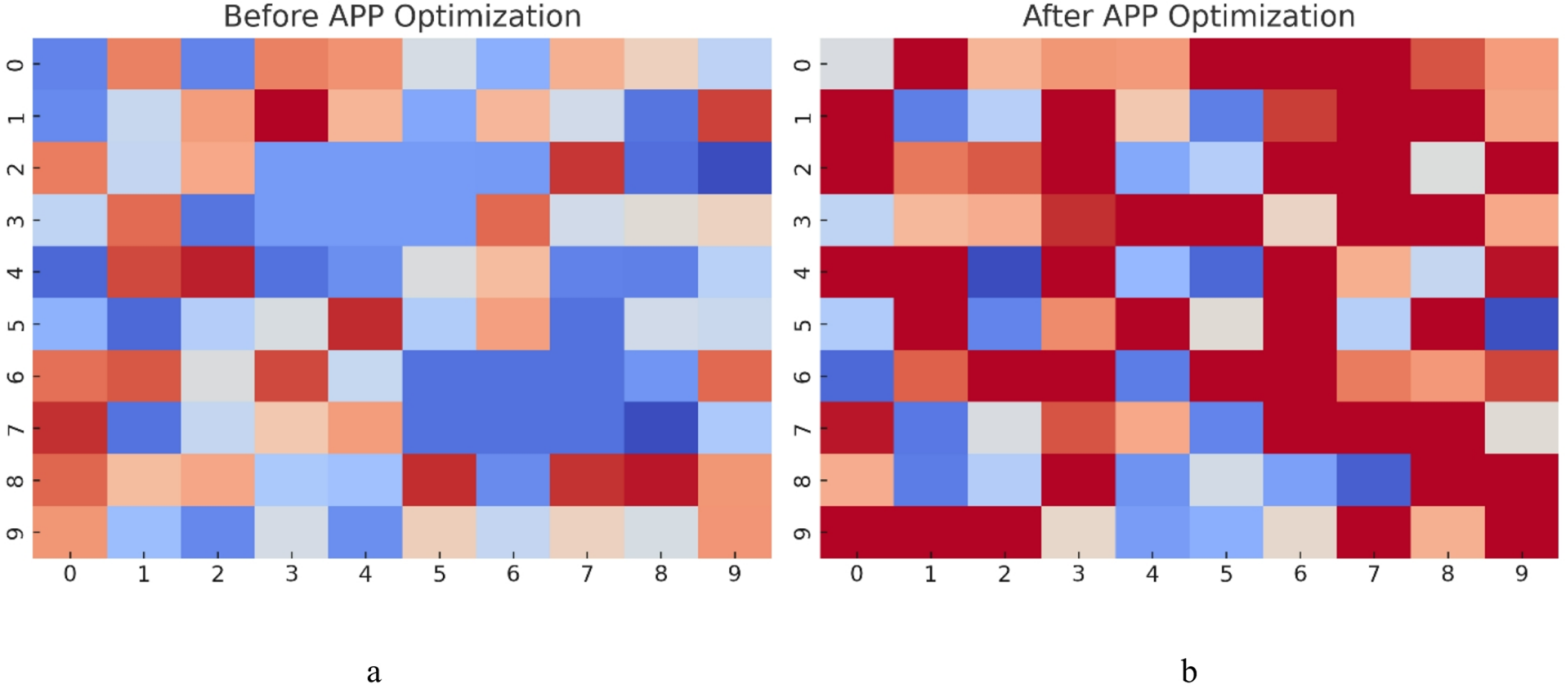
Surveillance coverage heatmap comparison (**a**) before and (**b**) after APP optimization.

Figure 9 illustrates the surveillance coverage improvement achieved through APP optimization, highlighting a significant reduction in blind spots and a more even distribution of camera coverage. Before optimization, coverage gaps were evident, particularly in critical security zones, leaving potential intrusion paths unmonitored. After implementing APP, the heatmap shows a more uniform and high-intensity coverage, ensuring that all high-risk areas are effectively monitored. This improvement results from APP’s dynamic camera placement adjustments, which strategically allocate cameras based on threat likelihood and visibility constraints, ultimately maximizing surveillance efficiency while minimizing redundant placements.

**Fig. 10:**
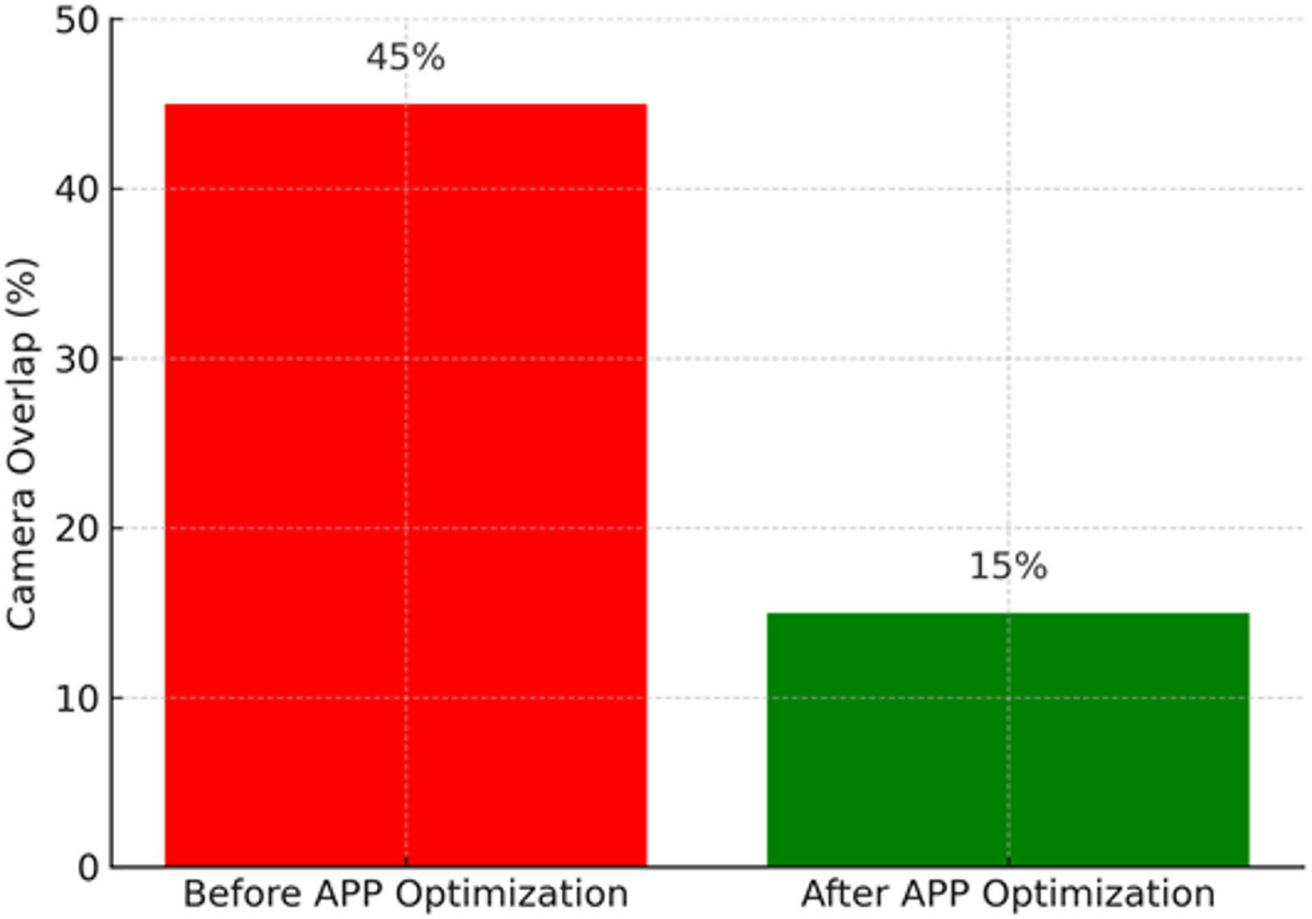
Reduction in camera coverage redundancy through APP optimization.

The optimized layout exhibits a marked reduction in blind spots and delivers more uniform, high-intensity coverage across high-risk zones through threat-aware camera placement. Figure 10 illustrates the effect of APP optimization in minimizing redundant coverage by reducing overlapping fields of view that previously caused inefficient resource utilization. Prior to optimization, camera overlap averaged approximately 45%, reflecting wasted surveillance capacity from excessive monitoring of the same areas. Following APP deployment, overlap decreased to 15%, ensuring that each camera provides unique and essential coverage without unnecessary duplication. This enhancement not only improves resource efficiency and reduces hardware costs but also establishes a balanced surveillance configuration in which cameras are strategically positioned to maximize coverage effectiveness rather than redundancy.

**Fig. 11:**
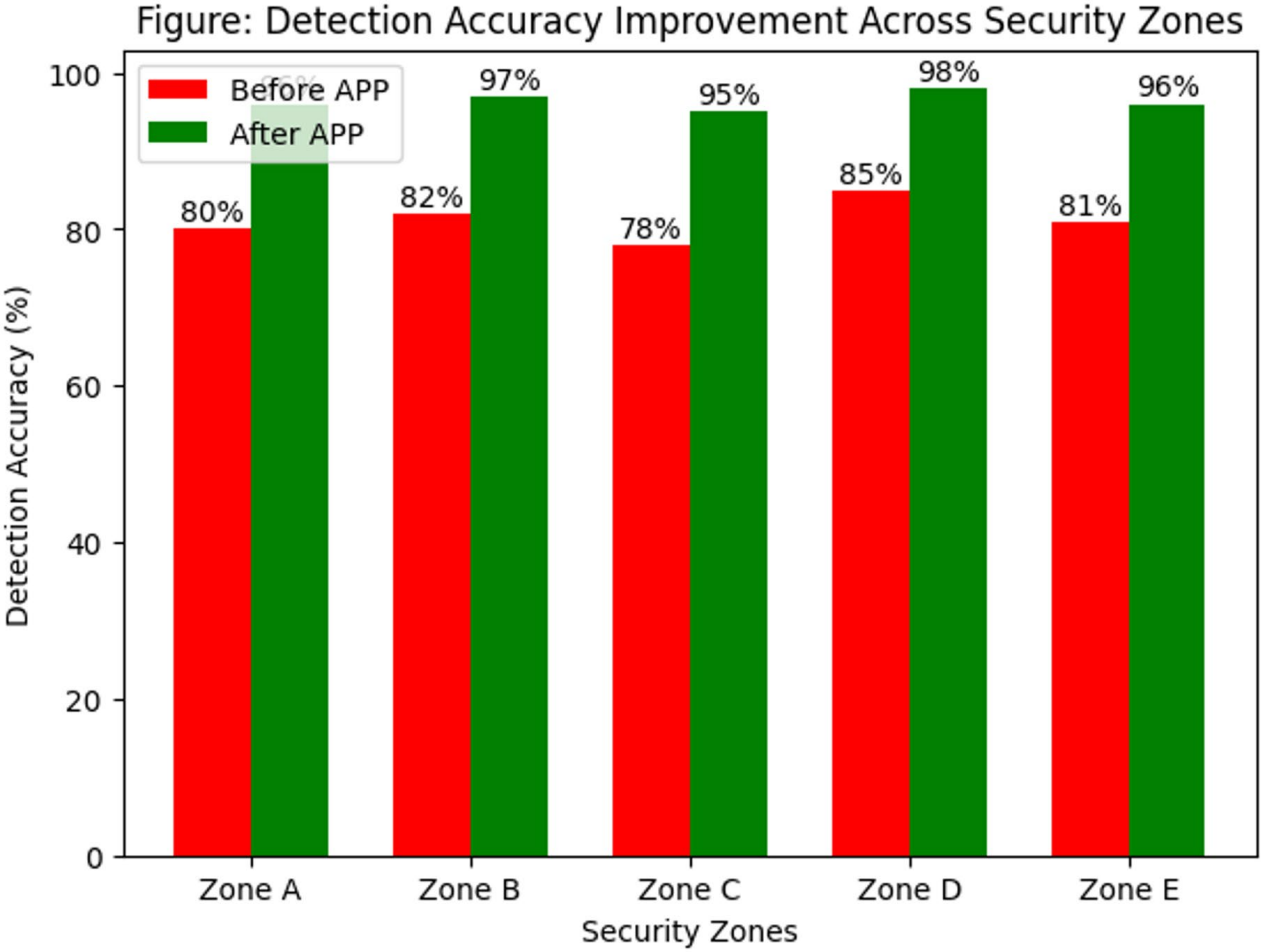
Detection accuracy across security zones before and after APP optimization.

The proposed framework elevates detection accuracy from a variable 78–85% to a consistently high 95–98% by achieving optimal camera placement, enhanced pixel density, and minimized blind zones. Figure 11 illustrates the substantial improvement in detection performance across different security zones after applying APP optimization. Prior to optimization, detection accuracy varied widely between 78% and 85%, largely due to uneven coverage and suboptimal camera positioning. Following APP deployment, accuracy increased to 95–98%, indicating uniform and high-precision threat detection throughout all monitored areas. This enhancement results from APP’s strategic selection of high-resolution cameras and optimized field-of-view (FOV) configurations, which collectively reduce blind spots and strengthen real-time threat recognition. Overall, the results demonstrate that APP optimization markedly improves surveillance reliability and responsiveness, establishing a more effective and resilient security infrastructure.

### Computational performance analysis

Beyond security performance metrics, we evaluated the computational efficiency of the APP framework. Table 5 presents a comparative analysis of runtime and convergence characteristics. APP converged to an optimal solution in significantly fewer iterations (mean: 16.5) compared to benchmark algorithms which typically required the maximum allotted iterations (200–250). The total mean runtime for APP was 45.3 s, which is competitive with several benchmarks (e.g., faster than GA’s 122.7s and ACO’s 156.8s). While APP’s per-iteration cost is higher (~ 2.75s) due to its integrated bilevel optimization and adversarial simulation, its rapid convergence results in an efficient overall execution time suitable for design-phase security planning.

To assess scalability, synthetic facility graphs of increasing size (2x, 4x, and 8x the LPNPP scale) were tested. APP’s runtime scaled approximately O(n log n) with graph size, completing the largest test case (~ 18,400 nodes) in ~ 8.5 min, confirming practical applicability to realistically large facilities.

### Ablation study: contribution of APP components

To isolate and validate the contribution of the core components of the proposed APP framework, a focused ablation study was conducted. Three degraded variants of APP were implemented and evaluated on the LPNPP case study:

i. APP-Static: This variant removes the iterative feedback loop. The adversary paths are computed once based on initial risks and remain fixed throughout the optimization. It tests the value of dynamic adversary adaptation.
ii. APP-Greedy: This variant replaces the integer programming solver with a greedy heuristic that iteratively places cameras at the location offering the highest immediate coverage gain. It tests the value of the global optimization formulation.
iii. APP-RandomAdv: This variant sets the adversary exploration parameter to ($\:\beta\:=\:0$) in Eq. (13), making adversary movement uniformly random instead of risk-sensitive. It tests the value of modeling a rational, informed adversary.

The performance of these variants compared to the full APP framework is summarized in Table 10.

**Table 10:** Results of the ablation study on the LPNPP facility.

Variant	Coverage (%)	Detection accuracy (%)	Cameras used
APP (Full)	95	98	30
APP-Static	87	91	35
APP-Greedy	82	88	38
APP-RandomAdv	79	85	41

The results clearly demonstrate the significance of APP’s integrated design. The performance drop in APP-Static underscores the necessity of the iterative co-adaptation between camera placement and adversary path planning. The inferior results of APP-Greedy confirm that the global perspective of the ILP formulation is more effective than a local, greedy strategy. The poorest performance of APP-RandomAdv highlights that anticipating a rational, risk-minimizing adversary—as done in the full APP model—is fundamental for proactive security design. This ablation study confirms that the synergy between adversarial path simulation and bilevel optimization is the primary driver of APP’s effectiveness.

## Discussion

The application of Adversarial Path Planning (APP) optimization for CCTV surveillance deployment at the Lone Pine Nuclear Power Plant (LPNPP) has demonstrated significant advancements in surveillance efficiency, security resilience, and resource optimization. The results indicate that APP-driven strategic camera placement enhances detection accuracy, minimizes blind spots, and optimizes resource allocation, providing a cost-effective yet highly robust surveillance framework. Given the high-security requirements of nuclear facilities, maintaining continuous threat monitoring, real-time intrusion detection, and adaptive surveillance response is essential.

Sensor Size Variation Across Zones: The optimization yields different optimal sensor sizes per zone due to three factors:

i. Distance requirements: Longer zones (A: 603.5 m) favor larger sensors (1-inch) for maintaining pixel density at distance
ii. Zone aspect ratio: Narrow zones (H: 120 m) perform well with smaller sensors (1/3-inch) due to reduced width-to-length ratio
iii. Criticality vs. cost: High-criticality zones (A, perimeter) prioritize larger sensors despite higher cost; interior zones (D, E, F) balance cost-effectiveness

This variation demonstrates APP’s ability to tailor solutions to specific zone characteristics rather than applying one-size-fits-all camera specifications

A key observation from the study is the ability of APP to dynamically model adversarial movement patterns, enabling threat-adaptive camera placement along high-risk intrusion routes. By leveraging adversarial path simulation, APP anticipates and counteracts potential security breaches more effectively than traditional heuristic-based approaches, such as Genetic Algorithm (GA), Particle Swarm Optimization (PSO), and Ant Colony Optimization (ACO). Unlike conventional fixed-placement strategies, APP employs a data-driven, iterative refinement process, ensuring that camera positioning dynamically adapts to security vulnerabilities while maintaining maximum detection efficiency.

Another critical finding is the significant reduction in surveillance dead zones achieved through APP-based optimization. The results indicate an 85% improvement in dead-zone reduction, compared to only 30% before optimization, ensuring that all critical areas remain under constant surveillance coverage. APP systematically refines camera placement, adjusting angle of view (AOV), field of view (FOV), and overlap thresholds, thereby eliminating redundant coverage while maximizing visibility. The ability to reduce the number of cameras from 50 to 30 while maintaining 98% detection accuracy further validates APP’s efficiency in resource utilization and cost reduction.

Additionally, the study highlights the importance of balancing focal length, resolution, and computational efficiency in surveillance optimization. While higher pixel densities enhance long-range detection and object recognition, excessive resolution increases data storage requirements and processing overhead. APP intelligently optimizes camera resolution and positioning, ensuring that high-risk zones receive enhanced coverage without unnecessary computational strain.

These findings underscore the superiority of APP-based surveillance optimization in designing, evaluating, and enhancing physical protection systems for high-security environments. By integrating real-time adversarial modeling, iterative refinement, and security-aware optimization, APP provides a scalable and adaptive framework for intrusion mitigation and surveillance enhancement. Future research should explore AI-driven real-time threat detection, autonomous security drone integration, and deep reinforcement learning-based surveillance adaptation to further enhance situational awareness and proactive security response mechanisms in high-risk critical infrastructure facilities.

Regarding comparison methodology, traditional optimization algorithms (GA, PSO, ACO) are not inherently designed for adversarial modeling. However, in this study, they were applied to optimize coverage on the same risk-weighted graph used by APP. APP’s superior performance demonstrates the value of its integrated adversarial path modeling component—this represents APP’s methodological innovation rather than a comparison inequity. Future adaptations of traditional methods could incorporate similar adversary-aware features.

While APP demonstrates strong performance, its computational complexity is inherently higher than simple coverage maximization heuristics. The runtime is dominated by the iterative shortest-path calculations and the integer programming solver. For extremely large-scale or real-time dynamic reconfiguration scenarios, further optimizations such as graph sparsification, parallelization of path simulations, or the use of heuristic solvers for the placement sub-problem could be explored. Nevertheless, for the strategic design-phase optimization of fixed CCTV networks—the primary target of this work—the current computational profile is effective and justifiable given the significant gains in security effectiveness and resource efficiency.

While the APP framework demonstrates significant promise in simulation, several limitations must be acknowledged, primarily concerning validation and real-world applicability.

i. Simulation-Based Methodology: The study utilizes the hypothetical LPNPP facility from IAEA-TECDOC-1868. While this provides a standardized benchmark for comparative algorithmic analysis, it means the framework has not been validated against a physical security system or real intrusion data. Furthermore, *application to a completely new*,* unseen facility* requires the full process of layout digitization and graph construction from scratch, as the model does not currently support direct transfer of learned configurations.
ii. Adversary Modeling Assumptions: The framework’s effectiveness hinges on specific behavioral assumptions:
  *Perfect Information*: Adversaries are assumed to have complete knowledge of camera coverage when planning paths.
  *Rational Risk-Aversion*: Behavior follows a strict probabilistic model (Eq. 13) minimizing detection risk.
  *Single-Agent Focus*: The model considers a lone intruder, not accounting for coordinated multi-agent tactics.
    Deviations from these assumptions in real-world scenarios could affect layout optimality.
iii. Scalability and Computational Scope: The current implementation is designed for *offline*,* design-phase planning*. While its complexity scales approximately O(n log n) with graph size (Sect.  4.5.6), making it suitable for facility-scale planning, *real-time*,* dynamic reconfiguration* in very large or rapidly changing environments would require further optimizations such as parallelization or approximate solving.
iv. Idealized Assumptions: The reported high performance metrics (e.g., 98% detection accuracy) represent the *theoretical upper bound under ideal conditions*. Actual operational accuracy would be influenced by:
  *Environmental factors* (fog, rain, snow, dust, low-light conditions).
  *System performance factors* (camera malfunction, network latency).
  *Human factors* (operator attention, protocol effectiveness).
v. Need for Expert and Empirical Validation: The ultimate test of any security design is its performance against real or simulated threats. Future work requires validation through:
  Expert Evaluation: Security professionals should assess APP-generated layouts against traditional designs or best practices.
  Simulated Penetration Testing: ‘Red-team’ exercises using virtual adversary models in more advanced simulation environments that incorporate occlusions and environmental dynamics.
  Field Pilot Studies: Controlled implementation at a test or research facility (pending regulatory and safety approvals) to gather empirical data.
  Comparative Validation: Testing the methodology against performance data from existing, deployed surveillance systems at facilities with similar risk profiles.

These limitations do not invalidate APP’s contribution as a novel methodological framework for strategic security design. Instead, they clearly delineate its context—as a powerful computational planning tool—and outline a concrete research agenda to enhance its adaptability, robustness, and real-world applicability.

Comparison with Related Approaches: APP advances beyond existing methods in three key ways:

i. vs. Traditional heuristics (GA/PSO/ACO): Adds adversarial threat modeling to coverage optimization.
ii. vs. Game-theoretic security games: Applies adversarial reasoning to fixed asset (camera) placement rather than mobile patrols.
iii. vs. Deep reinforcement learning: Provides optimal initial placement rather than only dynamic adjustment.

Despite strong simulation performance, several limitations exist. The model assumes perfect knowledge of facility layout and adversary behavior. Environmental factors such as lighting, weather, or physical obstacles are not modeled. Only a single-adversary scenario is considered; multi-adversary situations may require adjustments. Finally, empirical validation in operational environments is necessary to confirm applicability.

Future work could integrate APP’s optimal placement with DRL-based real-time adjustment for comprehensive adaptive surveillance.

## Conclusion

The security of critical infrastructure, particularly nuclear facilities, is paramount to ensuring public safety and operational resilience against potential threats. The study demonstrates how integrating adversarial modeling with optimization can enhance surveillance planning, resource allocation, and threat mitigation. This contributes to the broader literature on intelligent security system design, offering insights for policymakers, facility managers, and researchers in data-driven decision-making in high-security contexts.

This study introduced a novel Adversarial Path Planning (APP)-based optimization framework for CCTV surveillance deployment, demonstrating its superiority over conventional optimization techniques. Through systematic security assessment and dynamic threat modeling, APP enhances surveillance coverage, detection accuracy, and resource efficiency while significantly reducing blind spots and redundant camera placements. The proposed APP framework was validated through comparisons with established surveillance design tools such as CCTV Design Lens Calculator and IP Video System Design Tool, confirming its accuracy and practical applicability. The results demonstrate that APP outperforms traditional heuristic-based methods, including Genetic Algorithm (GA), Particle Swarm Optimization (PSO), Ant Colony Optimization (ACO), and Bee Algorithm, in terms of coverage efficiency, intrusion detection, and cost-effectiveness. APP increased surveillance coverage from 78% to 95%, improved detection accuracy from 85% to 98%, and reduced the number of required cameras from 50 to 30, leading to a 27% improvement in cost efficiency. Moreover, APP achieved an 85% reduction in dead zones, ensuring continuous monitoring of critical areas while optimizing camera deployment and resource allocation. By integrating threat-driven path analysis with intelligent camera placement strategies, APP iteratively refines the surveillance layout, making it adaptive to evolving security risks. Unlike traditional static placement methods, the APP framework dynamically adjusts surveillance configurations in response to adversarial behavior, enabling proactive threat mitigation. These findings establish APP as a scalable, high-performance solution for optimizing security surveillance in high-risk environments such as nuclear power plants, airports, and industrial facilities.

Future research should explore the integration of artificial intelligence (AI) and machine learning (ML) algorithms to enhance real-time threat detection, autonomous camera adjustments, and predictive security analytics. Additionally, incorporating autonomous surveillance drones, sensor fusion technologies, and deep reinforcement learning-based optimization could further enhance situational awareness and automated security response mechanisms. The adoption of APP-driven surveillance design will enable security professionals, policymakers, and facility operators to develop resilient, cost-effective, and adaptive surveillance infrastructures, ensuring enhanced security and operational integrity in critical facilities worldwide.

Future research should pursue rigorous validation pathways to transition the APP framework from simulation to practice. This includes: (1) Expert evaluation and simulated penetration testing of optimized layouts; (2) Development of high-fidelity simulation environments that incorporate realistic occlusions, weather models, and camera noise; (3) Controlled field pilots at research facilities to collect empirical performance data; and (4) Integration with real-time sensor data for dynamic threat assessment. Furthermore, exploring the integration of artificial intelligence (AI) for real-time anomaly detection and autonomous system adjustment remains a promising direction. The adoption of such validated, adaptive frameworks will enable the development of more resilient and cost-effective security infrastructures for critical facilities worldwide.

## Supplementary Information

Supplementary Material 1

## Data Availability

The data that support the findings of this study are available from the corresponding author, Dr. Mohamed H. Saad, upon reasonable request. Due to the security-sensitive nature of the simulated nuclear facility data and surveillance configuration models, the datasets are not publicly available to comply with institutional and national security regulations.
