# Supplementary material for: Adversarial path planning for optimal CCTV surveillance: a case study on nuclear facility security optimization
Source: Sci Rep. 2026 Apr 17;16:12697. doi: 10.1038/s41598-026-47647-8 (PMC13090357; doi:10.1038/s41598-026-47647-8)
Supplement: Supplementary file 1 — Supplementary Material 1 [file 41598_2026_47647_MOESM1_ESM.docx]

Figures S1 to S5 illustrate standard optical principles governing CCTV camera performance. They inform the camera parameter selection in the APP optimization but represent well-established relationships rather than novel contributions.

Figure S1: Relations between CCTV sensor width and height versus distance to object

Figure S2: Relations between CCTV sensor width and height versus dead-zone

Figure S3: Relations between CCTV focal length and distance to object

Figure S4: Relations between CCTV focal length and dead-zone

Figure S5: Relations between CCTV focal length and dead-zone
